# Improving the Cellular Accumulation of Folate-Conjugated Fully Chemically Modified siRNAs via 3′ Terminal Conjugation

**DOI:** 10.1021/acsomega.4c11519

**Published:** 2025-07-25

**Authors:** Keiichi Motosawa, Junko Iwano, Toshimasa Harumoto, Hayato Yabuuchi, Kentaro Hatanaka, Yasuo Koda, Toshiko Kubo, Hiroshi Kodaira, Keiji Uehara

**Affiliations:** Research Unit, Research Division, Kyowa Kirin Co., Ltd., 3-6-6 Asahi-machi, Machida-shi, Tokyo 194-8533, Japan

## Abstract

Folic acid (FA) conjugation is a validated tumor-specific delivery platform for small molecules. Although targeted delivery using FA-conjugated oligonucleotides, such as microRNA and small interfering RNAs (siRNA), has been reported, the performance of FA-conjugated fully chemically modified siRNA—a commonly used siRNA platform in clinical studies—remains unclear. To enhance the cellular accumulation of siRNA and subsequent gene knockdown (KD), we designed various FA–siRNA-based formats and evaluated their performance in folate receptor 1 (FOLR1)-expressing cells. Intracellular accumulation was enhanced by the conjugation of a substituent at the 3′ end of the antisense strand in FA–siRNA, which potentially stabilized the siRNA. Our study presents a promising approach for enhancing gene silencing in FOLR1-expressing cells.

## Graphical abstract

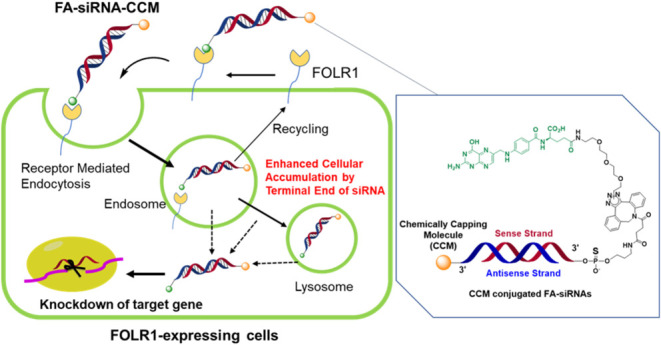

## Introduction

Synthetic oligonucleotides, such as antisense (ASOs) and small interfering RNAs (siRNAs), have great potential for targeting undruggable targets, for which classical small molecules or antibodies are not effective options.^1–5^ Various *N*-acetylgalactosamine-ligand-conjugated oligonucleotides have been approved for clinical use, particularly targeting disease-related genes in the liver.^6–9^ Ligand-conjugated oligonucleotides represent a simple therapeutic platform with great potential for extrahepatic delivery.^10–15^

Folate receptor-expressing cells are attractive experimental targets because the folate receptor is expressed on various tumors, kidney tubular epithelial cells, and macrophages.^16^ Multiple drug platforms targeting these cells, such as antibodies, antibody–drug conjugates, small-molecule-drug conjugates, and folic acid (FA)-conjugated nanocarriers/drugs, have been developed.^17,18^ In particular, FA conjugates are an excellent delivery platform in terms of structure simplicity and selectivity, with a high binding affinity for the folate receptor.^19–21^ Certain small-molecule-drug conjugates have entered the clinical stage.^19^

Willibald et al.^22^ first reported FA–siRNA conjugates that exhibited gene knockdown (KD) in folate receptor 1 (FOLR1)-expressing HeLa cells. Orellana et al.^23^ demonstrated *in vivo*-targeted delivery of microRNA (miRNA) to a tumor *via* FA conjugation. Rangasamy et al.^24^ and Orellana et al.^25^ enhanced RNA interference (RNAi) activity using an FA–oligonucleotide (miRNA or siRNA) conjugate with nigericin, a K^+^/H^+^ exchange reagent for endosomal escape. Salim et al.^26^ reported that, compared to siRNA, the centrally modified FA–siRNA conjugates showed enhanced gene KD activity. However, despite many reports on FA–oligonucleotide conjugates, few have described targeted delivery using fully chemically modified siRNAs, which have been widely adopted in clinical practice.^27,28^

As for the approaches for chemical modification of siRNAs, fluoro and *O*-methyl modifications at the 2′ position of the ribose sugar and phosphorothioate linkage (PS) are commonly used. These modifications improve the metabolic stability of siRNA *in vivo*, in turn enhancing gene KD.^4,29^ Recently, there have also been reports on chemical modifications that can be used to stabilize independently of the phosphorothioate linkage.^30^ In addition, the incorporation of phosphatase-resistant analogs, such as 5′-*E*-vinylphosphonate, at the 5′ end of the antisense strand could potentiate the gene silencing activity of siRNA *via* phosphate stabilization.^31–35^

On the other hand, conjugations to the multiple-terminal ends of siRNA enhance resistance to degradation by nucleases and also enable the attachment of multiple functional molecules such as ligands and membrane-permeable molecules. However, to the best of our knowledge, there are only a few studies of ligand-conjugated siRNA with multiple functional molecules utilized *via* the multiple-terminal end.^36^

In this study, we synthesized and evaluated FA–siRNAs with various molecules conjugated to the 5′ end of the sense strand or the 3′ end of the antisense strand (Figure 1). As additional chemical conjugation molecules (CCMs), we selected previously reported cell-penetrable molecules^37–40^ and non-cell-penetrable ones to confirm the effect of cytosolic transfer of conjugates. Regardless of whether the penetrable molecule was conjugated or not, the enhanced accumulation of siRNA in the cell was observed following additional terminal conjugation at the end of siRNA. Therefore, our study provides a useful reference for developing siRNA-based therapeutics targeting folate receptor-expressing cells.

**Figure 1:**
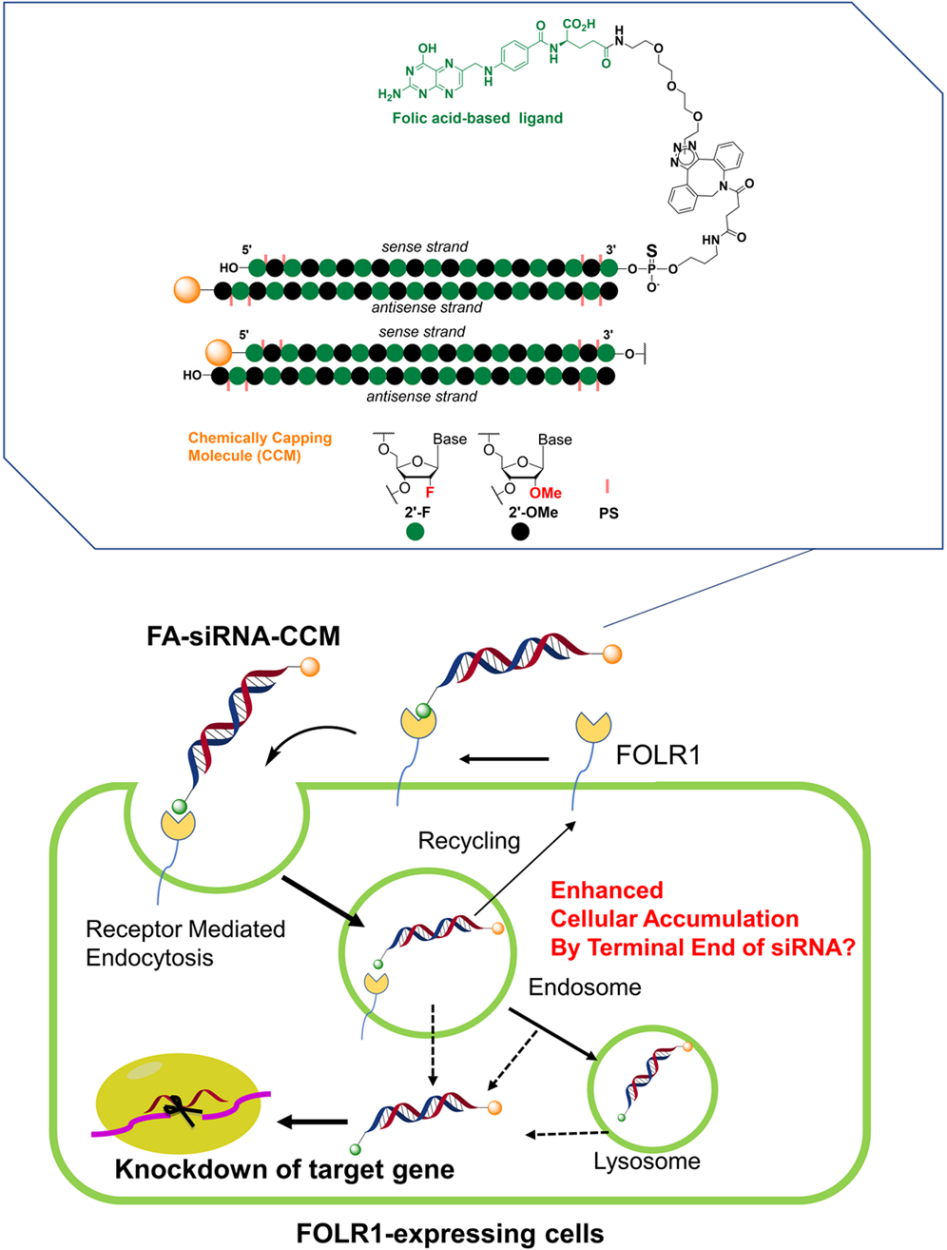
Schematic illustration of targeted delivery to FOLR1-expressing cells *via* FA–siRNA with chemical conjugation at the 3′ end of the antisense strand.

## Results and Discussion

### Preparation of FA–siRNA and Its Properties

We used siRNA targeting hypoxanthine phosphor-ribosyltransferase-1 (*HPRT1*) and β-2-microglobulin (B2M). The siRNAs were chemically stabilized by introducing 2′-O-methyl, 2′-deoxy-2′-fluoro, and phosphorothioate modifications (Table S1),^8,41^ resulting in the highest gene suppression activity that we have ever achieved across various cell types.^42,43^ Table 1 shows the structures of the terminally modified conjugates used in this study.

**Table 1:**
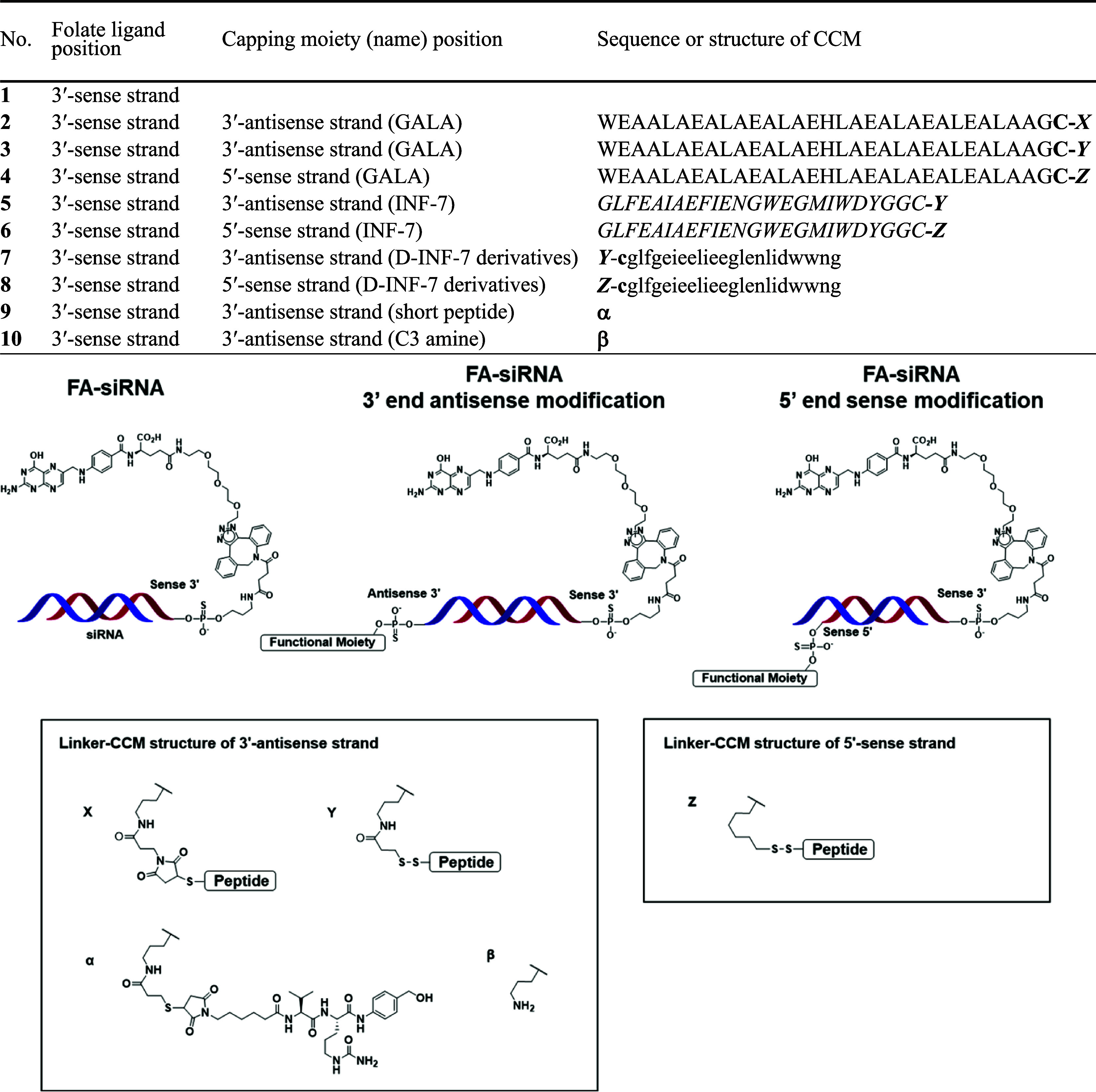
Terminal Modifications of FA–siRNA

The synthesis of FA–siRNA (**1**) is illustrated in Scheme S1. To verify the targeting effect *in vitro*, we evaluated the cellular accumulation and KD activity of FA–siRNA in FOLR1-expressing cells. We mainly used siHPRT1 targeting *HPRT1*. After treating KB cells with 10, 30, and 100 nmol/L of FA–siRNA (**1**) in FA-free medium, we quantified the cellular uptake of siHPRT1 using a stem-loop quantitative reverse transcription polymerase chain reaction (RT-qPCR).^44^ The intracellular levels of **1** were clearly higher than those of unconjugated siHPRT1, the negative control (Figure 2A). In KB cells, **1** exhibited a stronger KD effect on *HPRT1* mRNA than unconjugated siHPRT1 in FA-free medium (Figure 2B, siHPRT1_FA­(−) vs **1** _FA(−)). These results indicated that the KD activity corresponded to cellular accumulation. To confirm the ligand-dependent KD effect, we evaluated the KD activity of **1** in KB cells grown on 1 mg/L of FA-containing medium. Indeed, the gene KD activity in the presence of FA was lower than that under FA-free conditions (Figure 2B).

**Figure 2:**
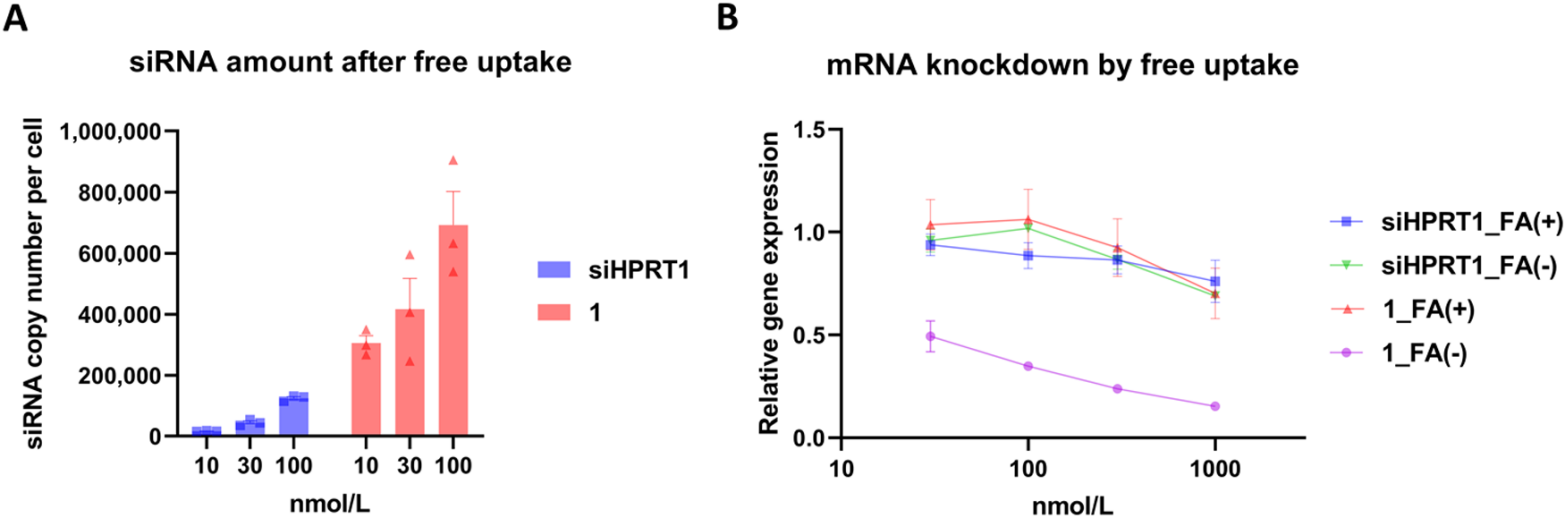
Cellular accumulation and gene knockdown properties of FA–siRNA. (A) The amount of siRNA after 24 h of free uptake in KB cells grown in FA-free medium. FA–siRNA and unconjugated-siRNA levels were analyzed *via* stem-loop RT-qPCR. (B) Gene silencing activities in KB cells. FA–siRNA and unconjugated siRNA were added to the cells for 3 days in the presence or absence of FA. *HPRT1* mRNA expression was quantified *via* RT-qPCR and normalized to ACTB mRNA expression. Data are expressed as the mean ± standard error (SE) from triplicate experiments.

### Cellular Accumulation and KD Effect of Various FA–siRNA Conjugate Formats

To investigate the enhancement of siRNA intracellular trafficking and gene KD effect, we designed and synthesized folate conjugates and confirmed their structure–activity relationship (SAR) by comparing KD activity or binding affinity. FA–siRNA was conjugated with a CCM at the terminal end of each strand for two reasons. First, the CCM at the terminal end can increase the stability of FA–siRNA against exonucleases. Second, the addition of CCM was expected to introduce a functional moiety such as an endosomolytic peptide to improve gene KD activity of FA–siRNA. We selected GALA, which is as a well-known endosomolytic peptide, to enhance the escape rate,^37^ and the HA2 domain of the influenza virus (INF7) as well as its mirror-image isomer (d-INF), which have been reported to enhance the KD efficiency on nucleic acid DDSs (nanoparticle and ligand conjugate).^45–47^ The scheme for selecting suitable conjugate formats is described in Figure 3A. All conjugates we used are listed in Schemes S2–S5.

**Figure 3:**
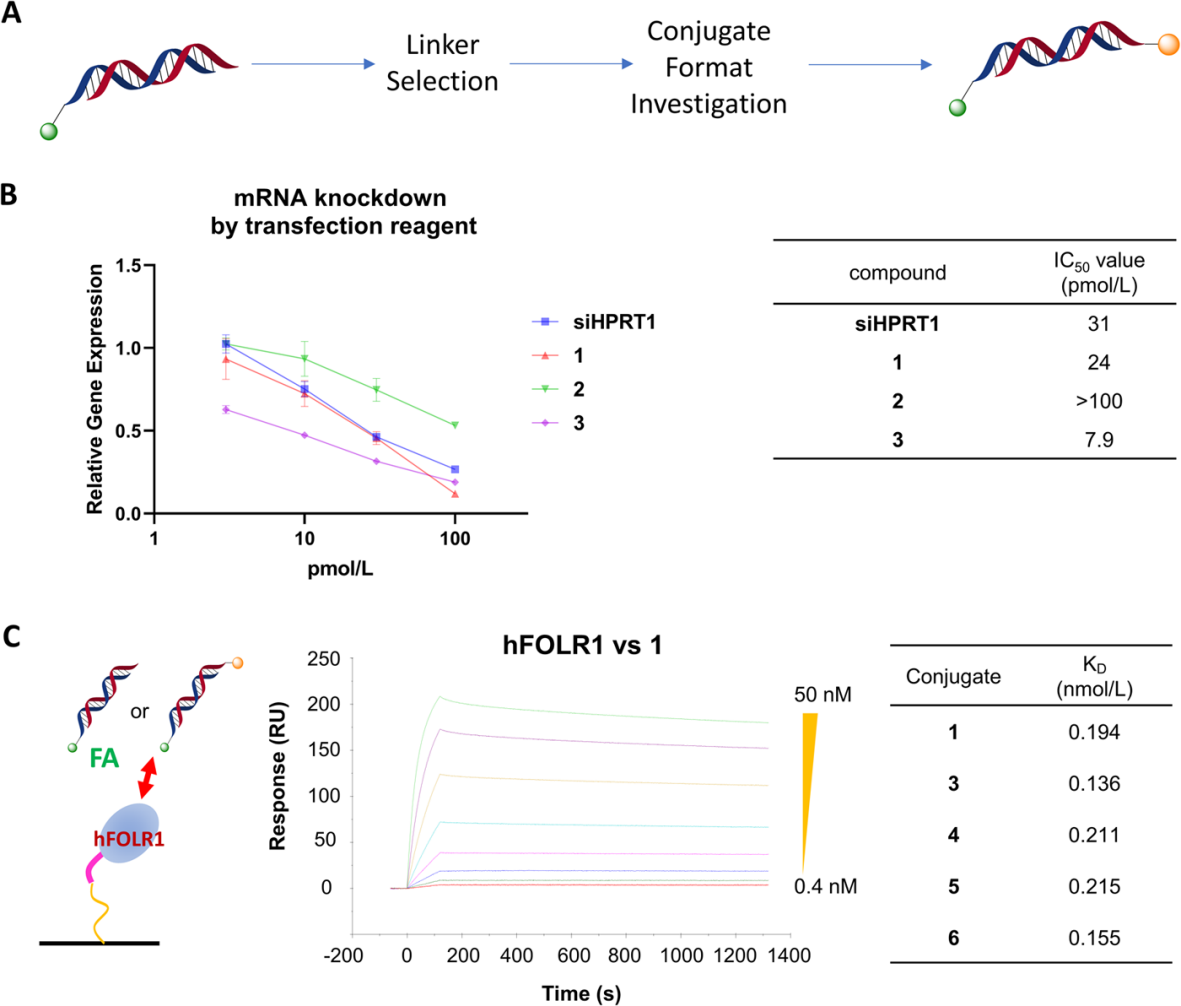
Selection of a suitable conjugate format using the transfection reagent and FOLR1-binding activities of conjugates. (A) Scheme for selecting a suitable conjugate format. (B) Gene KD effect of siRNA and conjugates in KB cells. The conjugates and siRNA were transfected using Lipofectamine RNAiMAX reagent for 1 day. *HPRT1* mRNA expression was quantified *via* RT-qPCR and normalized to ACTB mRNA expression. Right panel: IC_50_ values of siRNA and conjugates. Data are expressed as the mean ± SE of triplicate experiments. (C) Binding properties of FA–siRNA and its derivatives to human FOLR1 (hFOLR1). hFOLR1 was immobilized on a sensor chip, and each conjugate was added. Right panel: dissociation constant (*K*_D_) of each conjugate bound to hFOLR1.

First, in order to introduce large functional molecules as CCMs, we explored suitable linkers between the CCM and siRNA. This is because the silencing mechanism of siRNA requires that the antisense strand forms a complex with the Argonaute-2 protein. If the terminal structure becomes too large, there is a possibility of lower activity due to a decrease in complex formation ability (in particular, there is a possibility of modification to the 3′ end of the antisense strand). We designed cleavable/noncleavable linkers between the linker and oligonucleotide in order to introduce GALA—a membrane-fusible peptide, which has a relatively large molecular weight and membrane permeability function—into the 3′ end of the antisense. To confirm function within the cytoplasm, we evaluated KD activity using a transfection reagent (Figure 3B). The KD activity of FA–siHPRT1–GALA with a maleimide linker (**2**) was weaker than that of **1**. In contrast, the KD activity of FA–siHPRT1–GALA with a disulfide linker (**3**) was similar to that of **1**. When using FA unconjugated-siRNA–GALAs (siRNA–mal–GALA and siRNA–S–S–GALA) and siRNA, a similar tendency in the KD efficiency was noted (Figure S1). These results suggested that the introduction of an intracellularly cleavable linker around the 3′ end of the antisense strand can be functional in cytosolic conditions: the disulfide linker in **3** was cleaved under reducing conditions (the reaction was conducted under 10 mM glutathione/pH 7.4 in PBS buffer at 37 °C for 22 h; Figure S2).

To confirm the binding affinity between folate conjugates and the folate receptor, we conducted a surface plasmon resonance analysis using various conjugates (**1**, **3**, **4**, **5**, and **6**; the synthesis of these molecules is described in the Supporting Information). We observed only minor differences in the equilibrium dissociation constant (*K*_D_) values of each conjugate (Figure 3C).

Next, to investigate the impact of the conjugation site, we first tested the cellular accumulation of two types of FA–siRNA with GALA conjugates (**3** and **4**, GALA-modification position (3′ end of the antisense strand vs 5′ end of the sense strand)) in KB cells. We used CCM-unconjugated FA–siRNA (**1**) and siRNA as controls (Figure 4A). Both conjugates **3** and **4** showed enhanced KD activity compared to **1**, which corresponded to free uptake assay results (Figure 4B). We found that the increase in the cellular accumulation and gene KD activity was dependent on the conjugation position, as evidenced by the poorer KD activity of **4** compared to that of **3**. We further synthesized terminal conjugates with a folate ligand and a functional moiety at the 3′ end of the sense strand using a branched linker (FA–siHPRT1_ss3′brGALA; Figure S3A). The binding properties of FA–siHPRT1_ss3′brGALA to hFOLR1 were similar to those of **1** (Figure S3B; the *K*_D_ value of FA–siHPRT1_ss3′brGALA and **1** were 0.162 nmol/L and 0.194 nmol/L, respectively). Unlike **3** and **4**, FA–siHPRT1_ss3′brGALA did not show enhanced intracellular accumulation compared to that of **1** (Figure S3C). Similarly, the KD effect of FA–siHPRT1_ss3′brGALA was similar to that of **1** (Figure S3D). These results suggested that the enhanced intracellular accumulation and gene KD activities of FA–siRNA with chemical conjugation at the 3′ end of the antisense strand were related to the terminal modification of the oligonucleotides.

**Figure 4:**
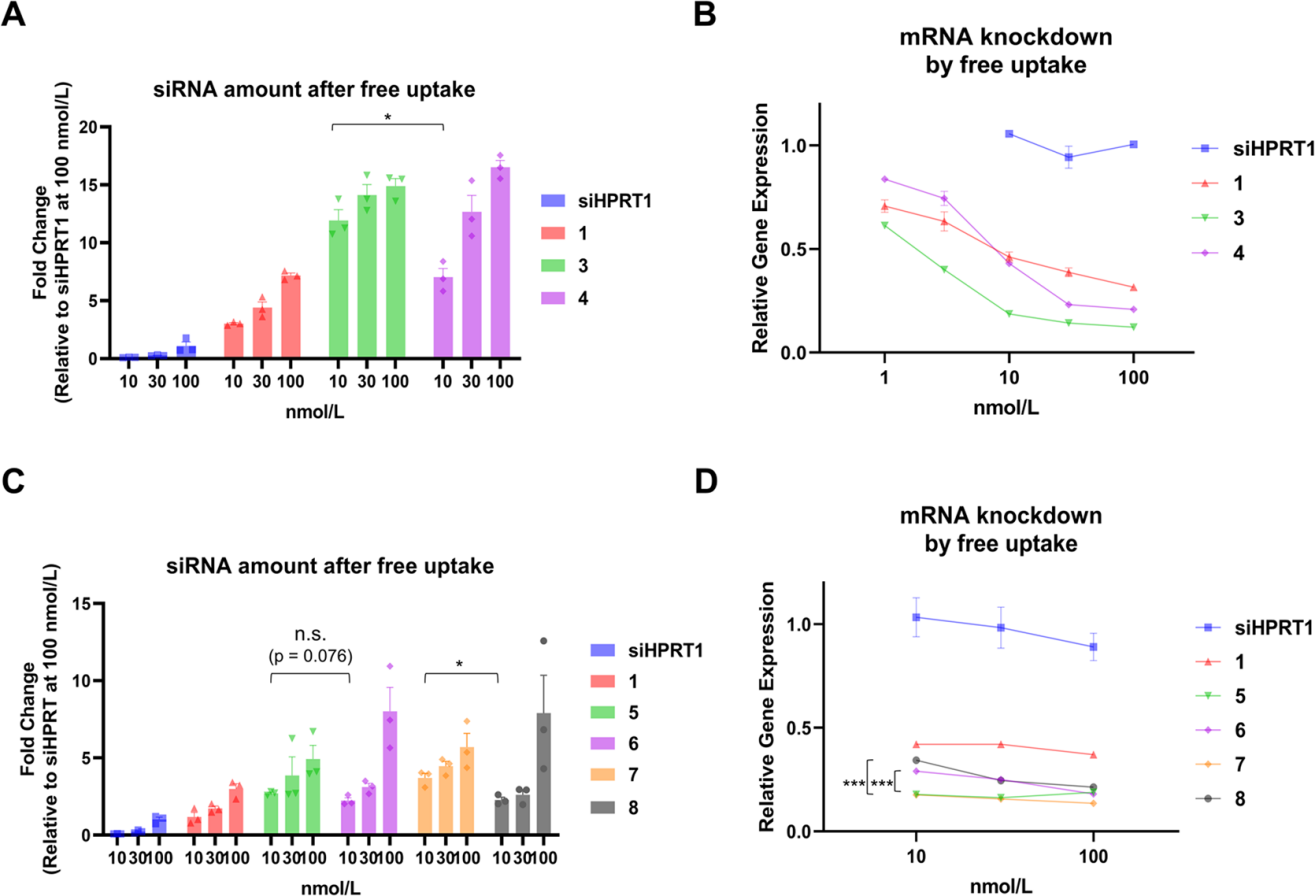
Intracellular accumulation and KD efficiency of FA–siHPRT1 with a functional moiety conjugated at the 3′ terminal end of the antisense or the 5′ end of the sense strand. (A) The amount of siRNA after 24 h of free uptake in KB cells. Amount of FA–siRNA and each conjugate in KB cells was analyzed *via* stem-loop RT-qPCR. (B) Gene KD activity in KB cells. FA–siHPRT1 (**1**), 3′-antisense GALA conjugate (**3**), 5′-sense GALA conjugate (**4**), or siHPRT1 were added to the cells for 3 d. *HPRT1* mRNA expression was quantified *via* RT-qPCR and normalized to the ACTB mRNA expression. (C) Amount of siRNA after free uptake in KB cells using fcmsiHPRT1, FA–siHPRT1 (**1**), 3′-antisense l *-*/d *-*INF7 conjugate (**5, 7**), and 5′-sense l *-*/d *-*INF7 conjugate (**6, 8**). The experimental conditions were the same as those in Figure 4A. (D) Gene KD activities in KB cells for siHPRT1, FA–siHPRT1 (**1**), 3′-antisense l *-*/d *-*INF7 conjugate (**5, 7**), and 5′-sense l-/d *-*INF7 conjugate (**6, 8**) *via* a free uptake assay. The experimental conditions were the same as in Figure 4B. Data are expressed as the mean ± SE from triplicate experiments. (*: *p* < 0.05, ***: *p* < 0.001, Student’s *t* test).

Furthermore, we investigated the effect of the different fusible peptide sequences (INF7 and d-INF peptides) to confirm the universality of the enhancement of intracellular accumulation and gene KD activity. Especially, we expected that d-INF made it possible further to enhance the intracellular accumulation and KD effect since it has been reported that d-peptides are generally more stable than non-d-peptides. Consistent with the GALA conjugates, 3′ end antisense and 5′ end sense conjugation with INF7 (**5** and **6**) or d-INF7 (**7** and **8**) showed enhanced intracellular accumulation and gene KD activity compared to **1** (Figure 4C,D). However, we did not confirm the clear difference in the intracellular accumulation and gene KD activity between INF and the d-INF peptide even though we had expected that the more stable d-INF would avoid peptide degradation, resulting in the stabilization of siRNA ends and an improved KD effect.

Based on our result, we confirmed that the enhanced KD activity of FA–siHPRT1 with 5′ end sense conjugation (**4**, **6**, and **8**) was consistent with the findings of previous reports,^35,36^ suggesting that the terminal conjugation of siRNA at the 3′ and 5′ ends of the sense strand enhanced gene silencing activity *via* oligonucleotide stabilization. Interestingly, the 3′ end antisense conjugates (**3**, **5**, and **7**) were more potent than the 5′ end sense conjugates (**4**, **6**, and **8**). However, there were no significant differences between the fusible peptide sequences used in this study in terms of intracellular accumulation and KD activities.

### Cellular Accumulation and KD Efficiency after Additional Conjugation at the 3′ End of the Antisense Strand

Despite the introduction of the endosomolytic peptides, little difference was observed in intracellular accumulation and KD activities. To clarify the requirement of endosomolytic function, we used short peptides and C3 amines as functional moieties, as these exhibit poor endosomal escape but appropriate steric hindrance ability for protecting against exonuclease degradation. Relative amounts of intracellular siRNA were evaluated, and IC_50_ values for each conjugate were calculated based on the expression of the target gene at four or five concentrations. Surprisingly, conjugates **9** and **10** showed the highest intracellular accumulation rates compared to **1** and **3** (Figure 5A) and, correspondingly, conjugates **9** and **10** had more potent KD activities than **1** (Figure 5B). In contrast to free uptake assay results, each conjugate showed a similar gene silencing activity in the transfection assay (Figure 5C). IC_50_ values for each conjugate were further correlated to the siRNA amount ratio after uptake (Figure 5D).

**Figure 5:**
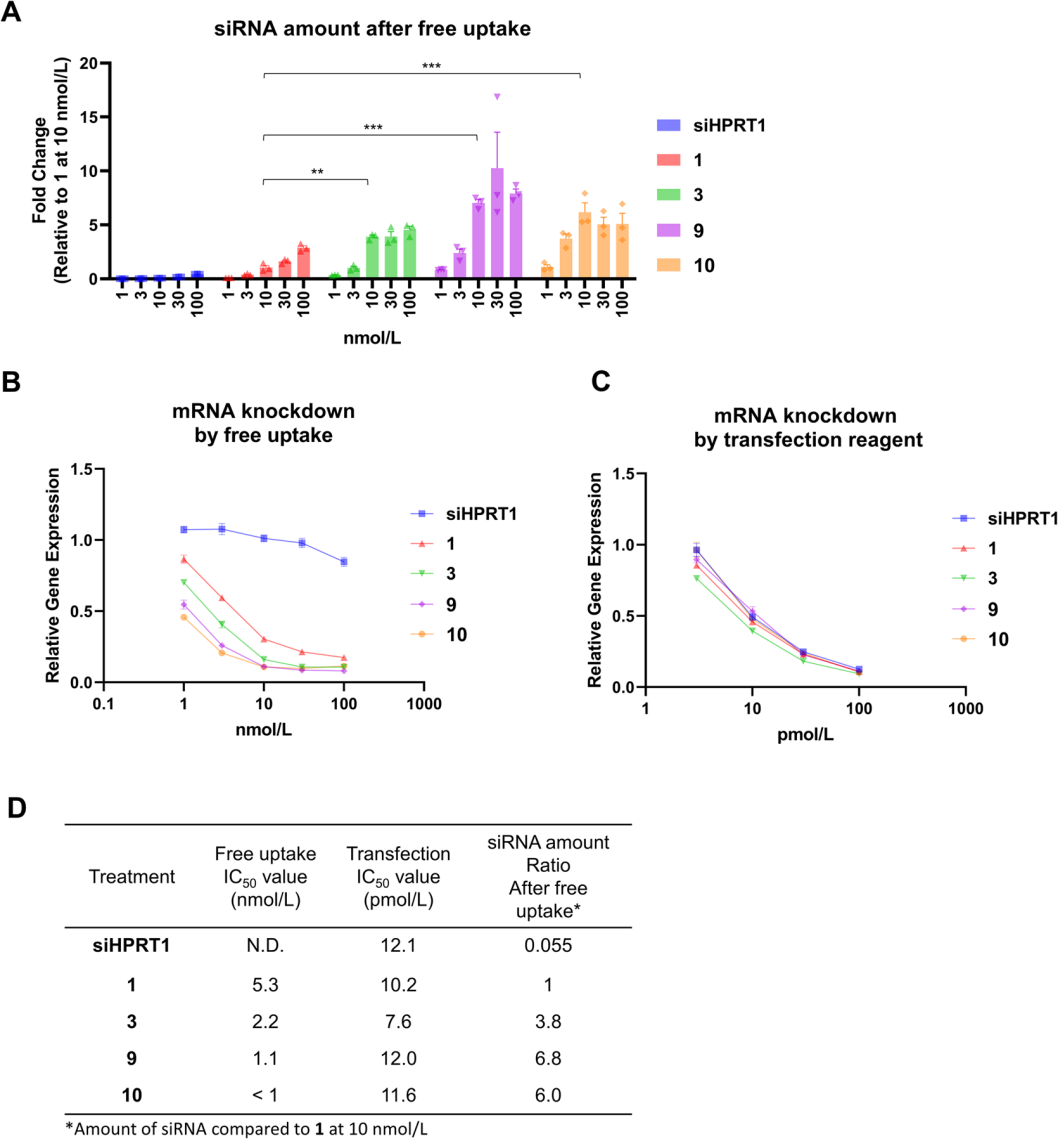
Intracellular accumulation and KD efficiency of FA–siHPRT1 conjugates with various modifications. (A) The amount of siRNA after free uptake in KB cells using siHPRT1 or conjugates. (**: *p* < 0.01, ***: *p* < 0.001, Dunnett’s test). (B) Gene KD efficiency based on the free uptake assay in KB cells. FA–siHPRT1 (**1**), 3′-antisense GALA conjugate (**3**), 3′-antisense short peptide conjugate (**9**), 3′-antisense C3 amine conjugate (**10**), or siHPRT1 were added to the cells for 3 d. *HPRT1* mRNA expression was quantified *via* RT-qPCR and normalized to ACTB mRNA expression. (C) Gene KD efficiency by siHPRT1 and conjugates assessed using transfection reagent. The conjugates and siRNA were transfected using Lipofectamine RNAiMAX reagent for 1 d. (D) Free uptake assay (B), IC_50_ values for the transfection assay (C), and siRNA ratios after free uptake at 10 nmol/L compared to **1**.

To elucidate the reason behind the greater intracellular accumulation values achieved *via* 3′end modification of the antisense strand, we tested the stabilization effect in the intracellular environment. First, we evaluated the stability of conjugates **1**, **3**, **9**, and **10** using SVPD as an exonuclease. Although conjugate **3** shows a slight tendency to decompose (details unknown), there is little change in siHPRT1 and conjugates **1**, **9**, and **10** from 0 to 24 h after SVPD addition (Figure S4). Therefore, we attempted another evaluation: the intracellular nucleic acid quantification in KB cells was compared by evaluating their uptake over time using conjugates **1**, **3**, **9**, and **10** (Figure S5A). In this experiment, a higher ratio of the 3′ end of antisense-modified conjugates **3, 9**, and **10** persisted for a long period compared to siHPRT1 or **1** (Figure S5B). This result suggested that conjugation at the 3′ terminal end of antisense strand enhanced the stabilization of conjugates.

### Validation of 3′ End Antisense Strand Modification Efficacy with Another Target

To confirm the beneficial effect of chemical conjugation at the 3′ end of the antisense strand, we tested the effects of an siRNA targeting the *B2M* gene. The 3′-antisense conjugates (**3-siB2M**, **9- siB2M**, and **10- siB2M**) tended to show enhanced cellular accumulation at low concentrations (10 nmol/L; Figure 6A,D). However, the free uptake assay showed a moderate decrease in KD activity compared to **1-siB2M** (Figure 6B,D; the IC_70_ value of **1-siB2M** was 8.5-, 2.4-, and 3.0-times lower than that of **3-**, **9-**, and **10-siB2M**, respectively). In addition, the KD activities of the 3′-antisense conjugates **3-siB2M**, **9-siB2M**, and **10-siB2M** were strongly reduced compared to that of FA–siB2M (**1-siB2M**; Figure 6B,D; the IC_70_ value of **1-siB2M** was 4.4-, 15.7-, and 8.9-times lower than that of **3-**, **9-**, and **10-siB2M**, respectively). These results indicated that the intracellular accumulation of siRNA increased following 3′-antisense modification even if we used a different target gene sequence. On the other hand, we observed a significant decrease in gene silencing activities for conjugates targeting the *B2M* gene. These results suggested that uptake *via* intracellular protein Argonaute-2 was lower. Therefore, to clarify the mechanism underlying enhanced KD efficiency, future studies should evaluate various fully chemically modified siRNAs targeting other mRNAs or several sites within the mRNA sequence. A mechanistic understanding has the potential to establish a universal structure for both the stabilization and potentiation of all siRNA sequences.

**Figure 6:**
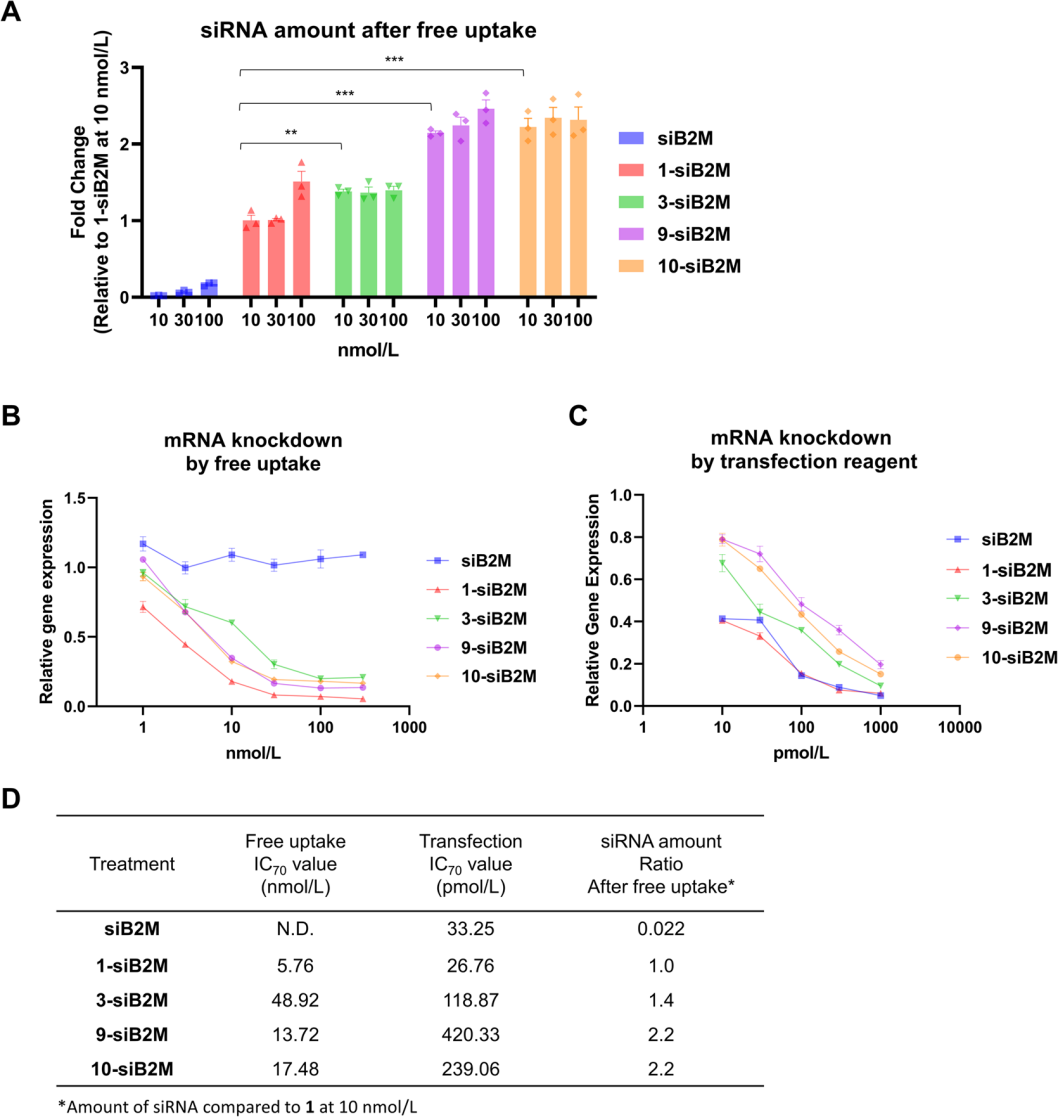
Confirmation of intracellular accumulation and KD efficiency based on sequence change. (A) The amount of siRNA after free uptake in KB cells using siB2M or conjugates (**: *p* < 0.01, ***: *p* < 0.001, Dunnett‘s test). (B) Gene KD efficiency based on the free uptake assay in KB cells. FA–siB2M (**1-siB2M**), 3′-antisense GALA conjugate (**3-siB2M**), 3′-antisense short peptide conjugate (**9-siB2M**), 3′-antisense C3 amine conjugate (**10-siB2M**), or siB2M were added to the cells for 3 d. *B2M* mRNA expression was quantified *via* RT-qPCR and normalized to the *ACTB* mRNA expression. (C) Gene KD efficiency of siB2M and conjugates assessed using transfection reagent. The conjugates and siB2M were transfected using Lipofectamine RNAiMAX reagent for 1 d. (D) Free uptake assay (B), IC_70_ values of the transfection assay (C), and siRNA ratios after free uptake at 10 nmol/L compared to **1-siB2M**.

Finally, we confirmed the dependence of KD efficiency on FOLR1 levels in three cell lines with high, intermediate, and no expression of FOLR1 (KB, OVISE, and ES-2 cell lines, respectively; Figure S6A). We also evaluated the gene KD activity of FA–siHPRT1 in OVISE and ES-2 cells. In OVISE cells, which express ∼20% less FOLR1 than KB cells, the KD activities of conjugates **3**, **9**, and **10** decreased (Figure S6B). In ES-2 cells, no KD efficiency was observed for conjugates **3**, **9**, and **10** (Figure S6C). These results indicated that KD efficiency was dependent on the FOLR1 expression on the cell surface.

## Conclusions

We investigated the intracellular accumulation of various FA-conjugated fully modified siRNAs. FA–siRNA with 3′-antisense modifications exhibited enhanced intracellular accumulation. Although FA–siRNA with 3′-antisense modifications also exhibited enhanced KD activity using mHPRT1-targeted siRNA, in the case of using mB2M-targeted siRNA, KD activity was reduced. Therefore, to clarify the mechanism underlying the enhanced KD efficiency, future studies should evaluate various fully chemically modified siRNAs targeting other mRNA or several sites within mRNAs. A mechanistic understanding has the potential to establish a universal structure for both stabilization and potentiation of all siRNA sequences. This will enable the development of a standardized siRNA platform with 3′ end modifications targeting various sequences, giving rise to improved nucleic acid-based drugs targeting FOLR1-low expressing cancers, kidney tubular epithelial cells, and macrophages.^48^ This study provides a useful framework for developing siRNAs against diseases caused by abnormal gene expression in FOLR1-expressing cells and new ligand-binding siRNA platforms.

## Materials and Methods

### Materials

All peptides shown in Table 1 were purchased from CosmoBio (Tokyo, Japan) and the TORAY Research Center (Kanagawa, Japan). Oligonucleotides used as the starting materials for conjugation (terminal functional group-conjugated passenger, passenger, fluorophore-conjugated guide, and guide strands) were obtained from Gene Design (Osaka, Japan).

The synthesis of chemical compounds and analytical data is provided in the Supporting Text. Schemes demonstrating the preparation of oligonucleotide conjugates are provided in the Supporting Information. The purity of FA–siRNA derivatives and mass spectrometry data of terminally modified oligonucleotides are provided in Tables S2 and S3, respectively. The analytical data for reversed-phase high-performance liquid chromatography and size exclusion chromatography are shown in the Analysis Data (Supporting Information).

### Binding Activity of FA–siRNA to FOLR1

A Biacore T100 (Cytiva, Tokyo, Japan) was used with HBS-EP+ Buffer 10× (Cytiva) as a running buffer. Recombinant human FOLR1 (hFOLR1; R&D Systems, Minneapolis, MN) was immobilized on the surface of the second flow cell of a Series S Sensor chip CM5 (Cytiva) using an Amine Coupling Kit (Cytiva). The binding affinity of each conjugate to hFOLR1 was measured by adding each conjugate to both flow cells. The flow rate for both association and dissociation was 30 μL/min. The chip surface was regenerated by adding 50 mmol/L of NaOH and 1 mol/L of NaCl for 20 s, followed by the addition of 3 mol/L of MgCl_2_ for 20 s. The concentration of each conjugate was 0.4–50 nmol/L. The *K*_D_ value was calculated to represent binding affinity using Biacore Insight Evaluation software (Cytiva).

### Human FOLR1-Expressing Cells

Human FOLR1-expressing cells (KB: JCRB9027, OVISE: JCRB1043) were directly purchased from the Japanese Collection of Research Bioresource Cell Bank (Osaka, Japan). Human FOLR1-negative cells were directly purchased from the American Type Culture Collection (ES-2: CRL-1978, Manassas, VA). hFOLR1 expression was quantified using a QIFIKIT instrument (Dako, Glostrup, Denmark).

### RNA Quantification

KB cells were seeded in each well of a 96-well plate in FA-free medium (Thermo Fisher Scientific, Waltham, MA) and incubated for 1 d with each of the experimental compounds. The cell lysates were prepared using a SuperPrep Cell Lysis & RT Kit for qPCR (Toyobo, Osaka, Japan) or TaqMan Gene Expression Cells-to-CT Kit (Thermo Fisher Scientific). cDNA was synthesized from the extracted RNA and subjected to qPCR as previously described.^44^ Cellular miR-16 expression was assessed using a TaqMan MicroRNA assay (Code No. 000391) (Thermo Fisher Scientific), and siRNA copy numbers were calculated by substitution of the CT value in the regression equation of the standard curve. To calculate the relative cellular siRNA levels, the amount of siRNA was normalized to that of miR-16. Each value is relative to that of 100 nmol/L of siRNA.

### Gene KD Efficiency

For the transfection assay, KB cells were seeded in each well of a 96-well plate and transfected using Lipofectamine RNAiMAX reagent for 1 d according to the manufacturer’s protocol.

For the free uptake assay, KB cells were seeded in each well of a 96-well plate in FA-free medium and incubated for 1 d. Each experimental compound was added to the cells for 3 d. The preparation of cDNA, measurement of *HPRT1* and *B2M* transcript levels, and quantification of the relative mRNA expression were conducted as previously reported.^44^

### Data Analysis

GraphPad Prism version 9 (San Diego, CA) was used for plotting the data. To calculate IC_50_ values, we fitted the dose–response curves for percent activity using a four-parameter logistic equation with XLfit 5.5.0.5 (IDBS, Boston, MA). Dunnett’s test was used to compare differences between the control sample and each compound, while Student’s *t* tests were used to assess differences between the two groups. Results with *P* values less than 0.05 were considered statistically significant.

## Supplementary Material



## Data Availability

All data generated or analyzed during this study are included in this published article and its Supporting Information files.

## Notes

Materials, oligonucleotide conjugation; preparation of FA–siRNA (1), preparation of FA–siRNA with the 3′ end antisense strand modification, preparation of FA–siRNA with the 5′ end sense strand modification, preparation of FA–siRNA modified at the 3′ end of the antisense strand using a branched linker; siRNA sequences used in this study, purity of FA–siRNA derivatives; the gene knockdown efficiency of siRNA and conjugates, stability of compound 3 with a disulfide linker in the reduction condition, the stability of the conjugates and fully chemically modified siRNA; and analytical data (PDF)
