# Supplementary material for: Improving the Cellular Accumulation of Folate-Conjugated Fully Chemically Modified siRNAs via 3′ Terminal Conjugation
Source: ACS Omega. 2025 Jul 25;10(30):32744–53. doi: 10.1021/acsomega.4c11519 (PMC12332678; doi:10.1021/acsomega.4c11519)
Supplement: Supplementary file 1 [file ao4c11519_si_001.pdf]

Supplementary Information

Improving the Cellular Accumulation of Folate-Conjugated Fully Chemically Modified siRNAs via 3' Terminal Conjugation

Keiichi Motosawa, Junko Iwano, Toshimasa Harumoto, Hayato Yabuuchi, Kentaro Hatanaka, Yasuo Koda, Toshiko Kubo, Hiroshi Kodaira, and Keiji Uehara*

Research Unit, Research Division, Kyowa Kirin Co., Ltd., 3-6-6 Asahi-machi, Machida-shi, Tokyo 194-8533, Japan

*Correspondence:

Research Unit, Research Division, Kyowa Kirin Co., Ltd. 3-6-6 Asahi-machi, Machida-shi, Tokyo 194-8533, Japan

Email: keiji.uehara.vk@kyowakirin.com; Phone: +81-80-7200-7306; Fax: +81-3-5205-7153

This PDF file includes:

- Supplementary Text
- Scheme S1
- Scheme S2
- Scheme S3
- Scheme S4
- Scheme S5
- Table S1
- Table S2
- Table S3
- Figure S1
- Figure S2
- Figure S3
- Figure S4
- Figure S5
- Figure S6
- Analytical Data
- Supplemental References

Supplemental Text

Materials

RNase-free water was purchased from Otsuka (Tokyo, Japan); triethylammonium acetate (TEAA) buffer, Sigma-Aldrich (St. Louis, MO, USA); NaCl solution, 1-[bis(dimethylamino)methylene]-1*H*-1,2,3-triazolo[4,5-b]pyridinium 3-oxide hexafluorophosphate (HATU), FUJIFILM Wako Pure Chemical Corporation (Osaka, Japan); phosphate-buffered saline (PBS), Nacalai Tesque (Kyoto, Japan); dibenzocyclooctyne-*N*-hydroxysuccinimidyl ester (DBCO-NHS), Click Chemistry Tools (Scottsdale, AZ, USA); succinimidyl 3-(2-pyridyldithio) propionate (SPDP), Dojindo Laboratories (Kumamoto, Japan); folate-TEG-azide, Berry & Associates (Dexter, MI, USA); and MC-Val-Cit-PAB-OH, Broad Pharm (San Diego, CA, USA).

Oligonucleotide conjugation

The conjugates were obtained as illustrated in Schemes S1-S4. Oligonucleotides were purified via preparative ion-pair reversed-phase high-performance liquid chromatography (IP-RP-HPLC) using a Prominence system (Shimadzu, Kyoto, Japan) with an X-Bridge Prep column (C18, 5 µm, 10 × 100 mm; Waters, Milford, MA, USA) and 0.1 M TEAA (pH 8.0) buffers with an acetonitrile gradient. Pure fractions were desalted using NAP columns (GE Healthcare UK, Little Chalfont, UK). The concentration of the desired oligonucleotide was determined using an Amicon Ultra device (MWCO 3 kDa; GE Healthcare). Single-strand oligonucleotide masses and purities were verified via liquid chromatography–tandem mass spectrometry (LC–MS/MS) on an Agilent 6530 Accurate-Mass system using linear gradients of methanol in water containing 9 mM Et_3N and 100 mM 1,1,1,3,3,3-hexafluoroisopropanol. The concentrations of oligonucleotides were quantified using Dropsense (Trinean, Gentbrugge, Belgium). The purity of the desired siRNA conjugates was determined using size exclusion chromatography (SEC-HPLC) with an X-Bridge BEH 200Å column (7.8 × 300 mm, Waters) and PBS/ acetonitrile (*v/v* = 3/7). The purity and mass of each oligonucleotide are summarized in Tables S1 and S2.

<u>Preparation of FA–siRNA (**1**)</u>

Scheme S1. Preparation of FA-siRNA (1)

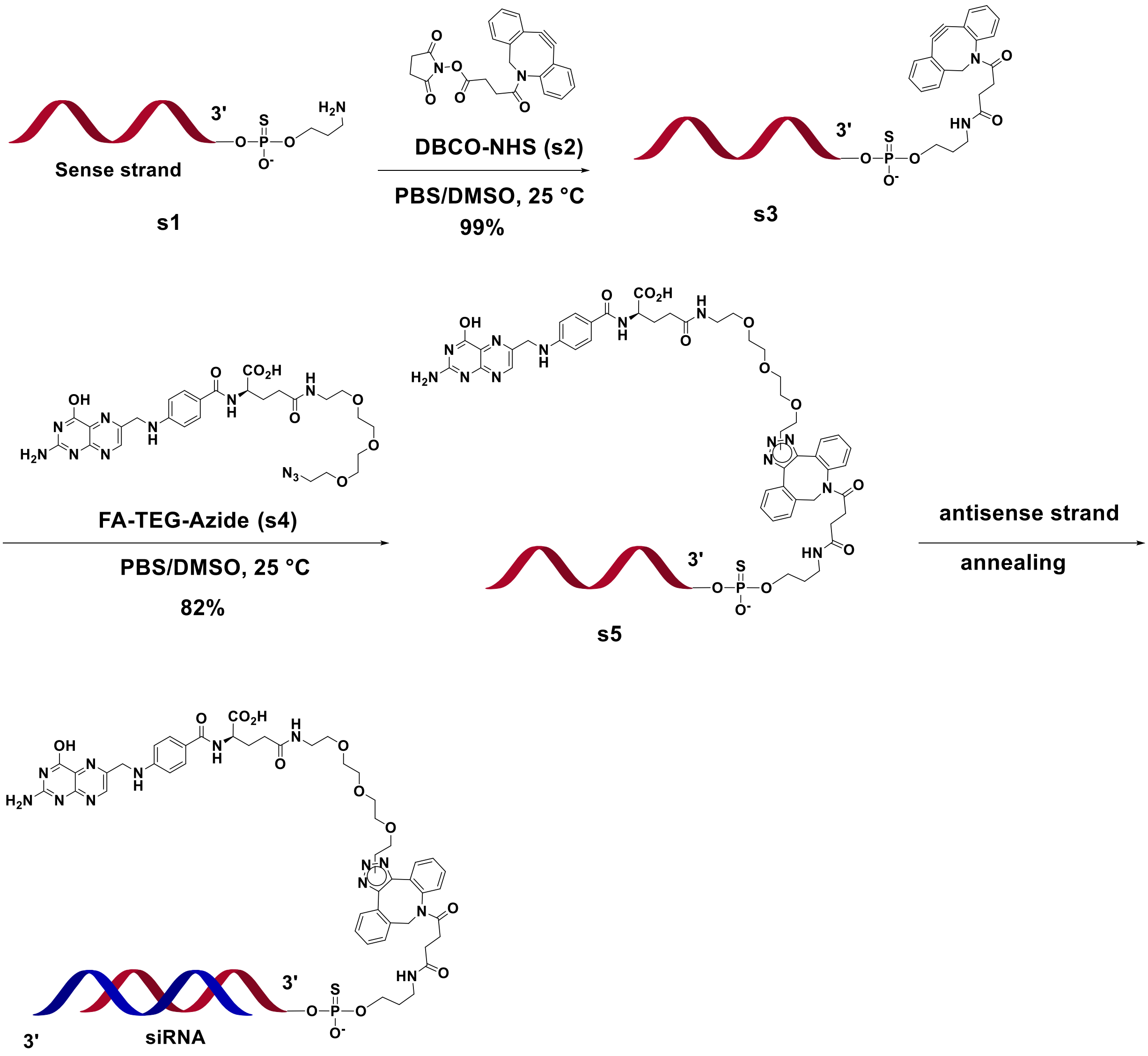

FA-siRNA (1)

The FA-conjugated oligonucleotides were synthesized as illustrated in Scheme S1. A reaction of the C3 amine-modified sense strand (**s1**) with DBCO-NHS (**s2**) in PBS/DMSO at 25 °C for 3 h yielded DBCO-modified RNA (**s3**). Conjugate **s3** was separated and buffer-exchanged using a NAP-5 column with PBS. Conjugate **s5** was obtained by reacting conjugate **s3** with folate-TEG-azide (**s4**) in PBS/DMSO at 25 °C overnight. After completion of the reaction, which was monitored using LC–MS/MS, the reaction mixture was purified via RP-HPLC. The pure fractions were desalted using NAP columns and concentrated using the Amicon Ultra device.

3′ end antisense strand modifications (**s8–s12**)

Scheme S2. Preparation of 3’ end-modified antisense strands.

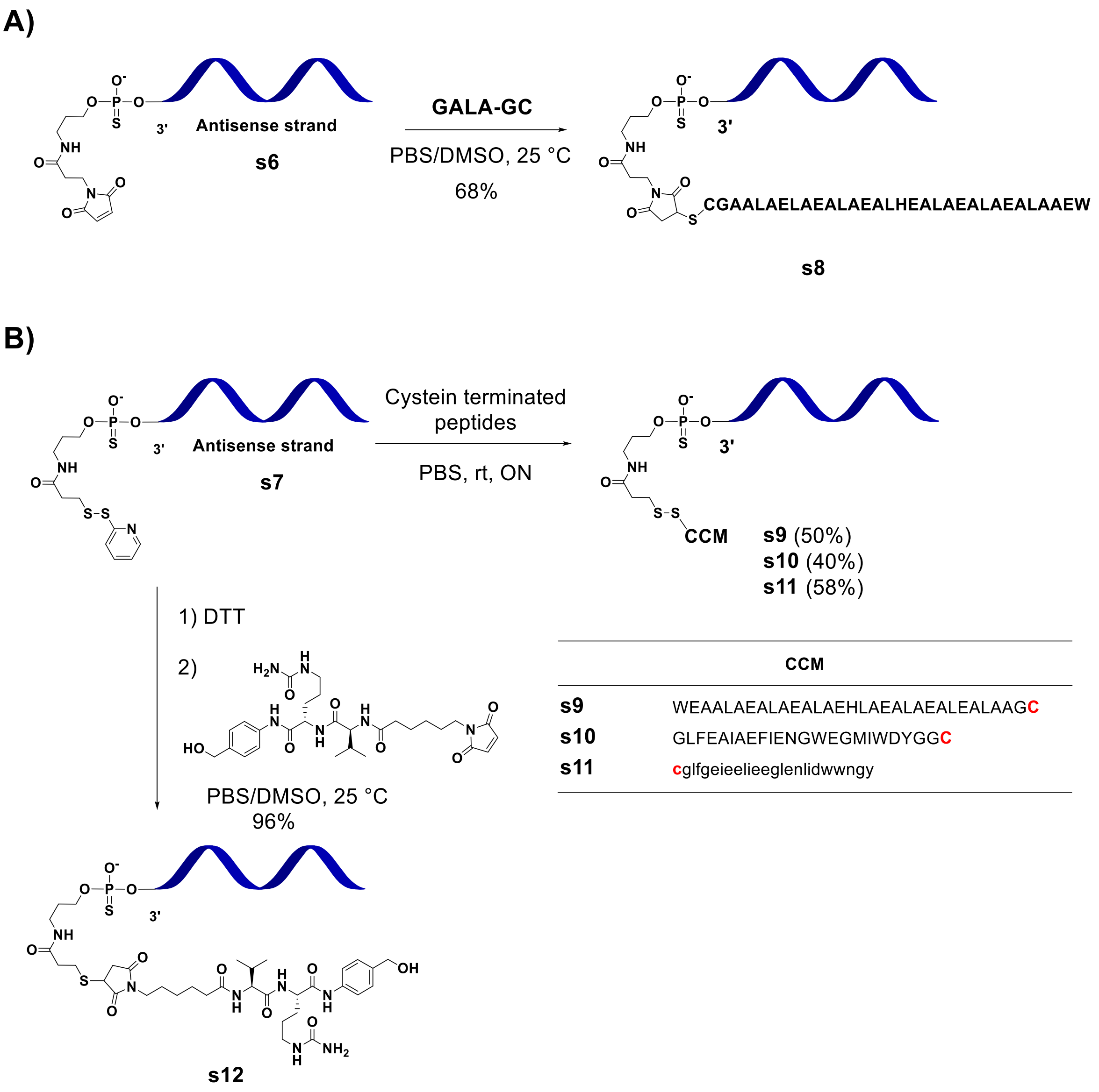

	CCM
**s9**	WEAALAEALAEALAEHLAEALAEALEALAAGC
**s10**	GLFEAIAEFIENGWEGMIWDYGGC
**s11**	cglfgeieelieeglenlidwwngy

Conjugates **s6–s11** were synthesized as previously described[1]. For the synthesis of **s12**, we used **s7** as a starting compound. Conjugate **s7** was added to 1 M DTT in PBS and reacted at 25 °C for 1 h, yielding as-3'-thiol-modified-RNA (as-3'-thiol-C3-RNA). After completion of the reaction, the reaction mixture was separated and buffer-exchanged using a NAP-10 column with PBS. We added as-3'-thiol-modified-RNA to MC-Val-Cit-PAB-OH in PBS/DMSO for a 3-h incubation at 25 °C to yield conjugate **s12**. After completion of the reaction, the mixture was purified by IP-RP-HPLC with the X-Bridge Prep system and 0.1 M TEAA buffer/acetonitrile.

FA–siRNA with 3' end antisense modification (**2, 3, 5, 7, 9, 10**)

Scheme S3. Preparation of FA-siRNA with 3' end antisense strand modification.

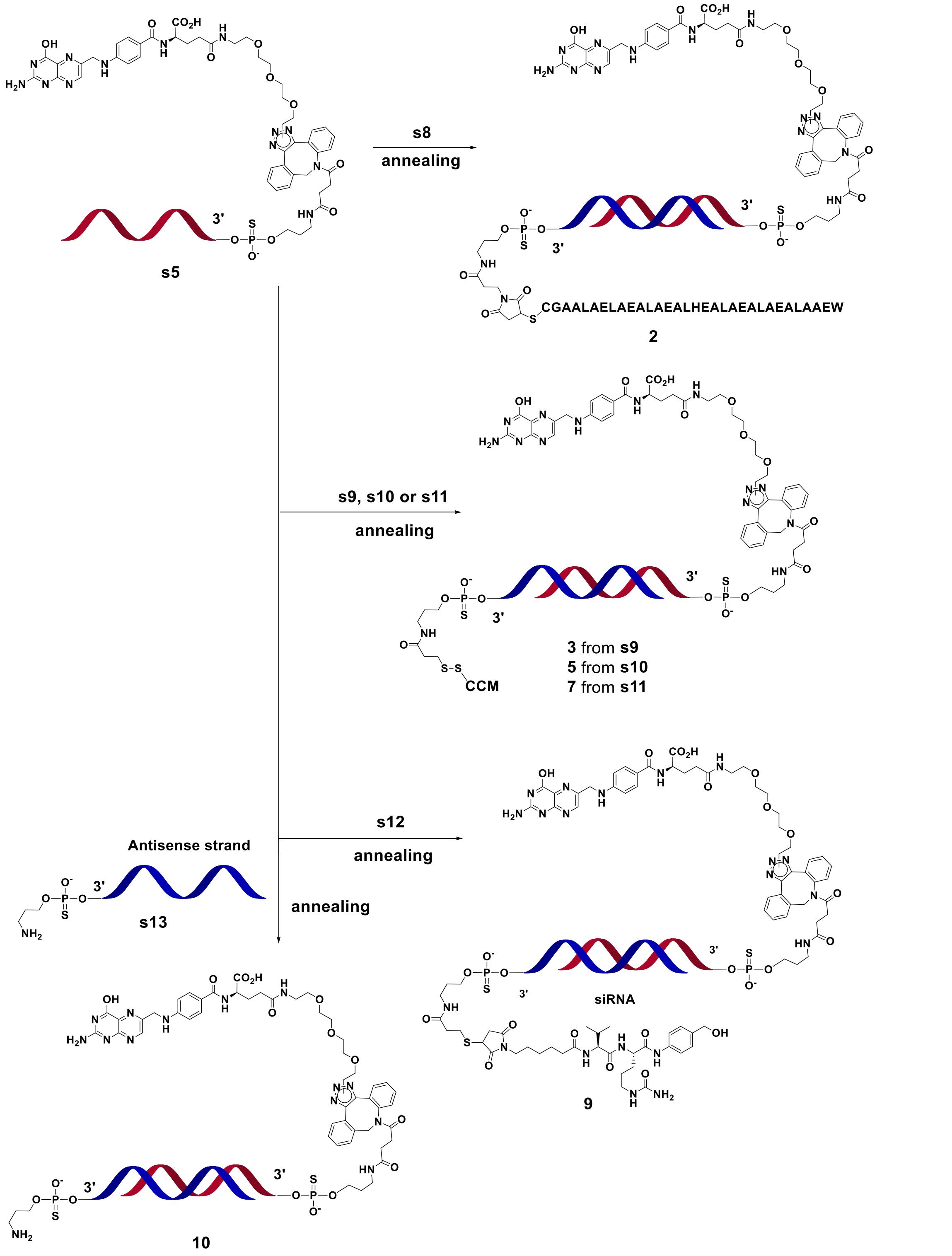

FA–siRNA and FA–siRNA_3'asCCMs were prepared from the annealing of conjugate **s5** with the corresponding antisense strand at 70 °C for 10 min. After cooling to room temperature, the purity

of the desired conjugates (modified folate-siRNAs) was determined using SEC-HPLC with the X-Bridge BEH 200Å column (7.8 × 300 mm, Waters) and PBS/acetonitrile (*v/v* = 3/7).

FA–siRNA with 5' end sense modification (**4, 6, 8**)

Scheme S4. Preparation of FA-siRNA with 5 'end sense strand modification.

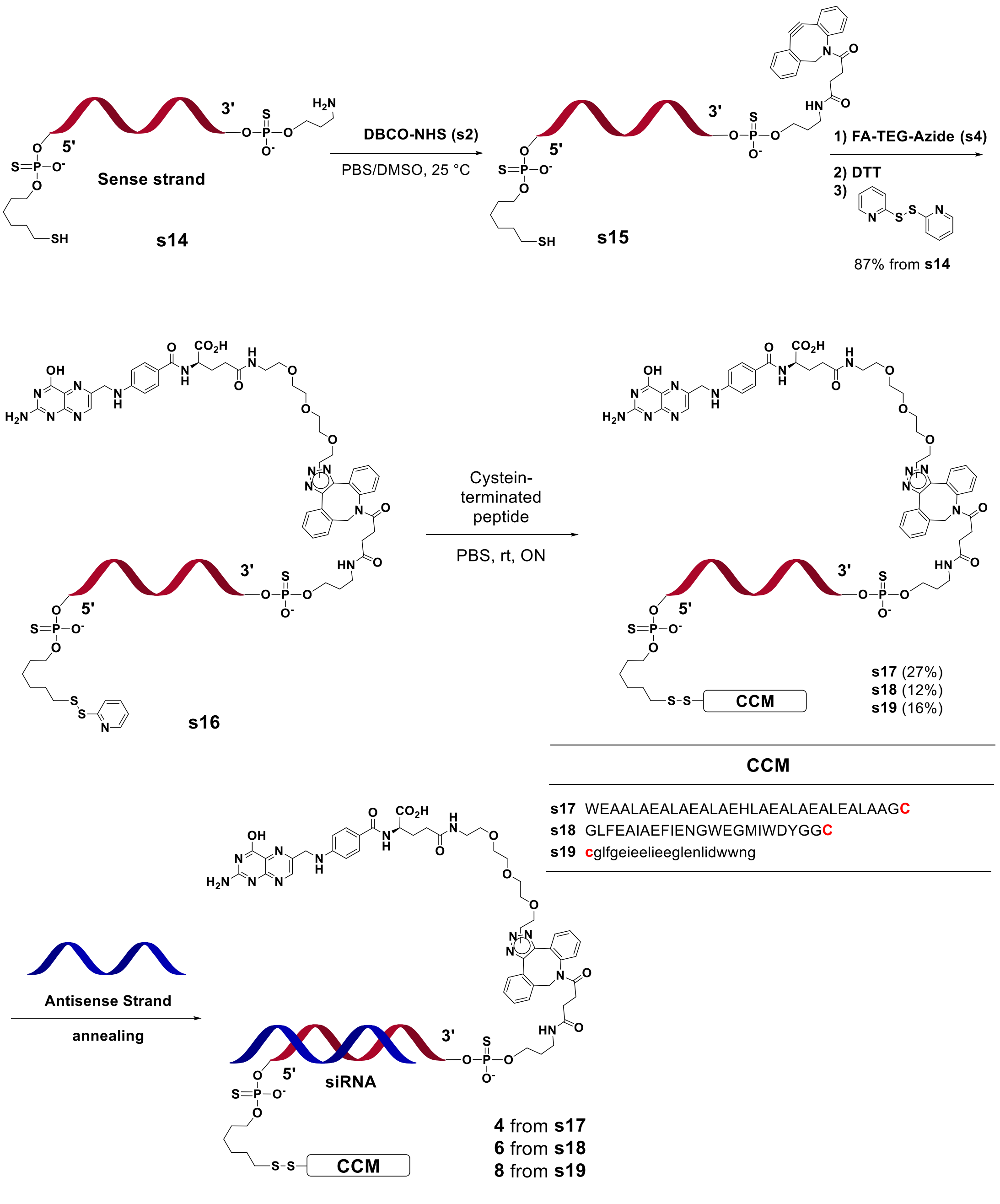

FA-fcmsiRNA_5'ssCCM

The reaction of conjugate **s14** with DBCO-NHS in PBS/DMSO at 25 °C for 3 h yielded conjugate **s15** and a byproduct (3′- and 5′-sense strand di-DBCO-modified conjugates). Adding ammonia

solution to the reaction mixture yielded only conjugate **s15**, which was separated using NAP-10 columns with PBS. After concentration, conjugate **s15** was added to folate-TEG-azide (**s4**) in DMSO/PBS at room temperature and left overnight. After completion of the reaction, 1 M DTT solution was added to the reaction mixture and left at 4 °C for 3 h. The mixture was separated, buffer-exchanged, and concentrated. Next, the intermediate was added to di(2-pyridyl) disulfide in PBS/DMSO at 25 °C for 3 h to yield the 5′-sense strand SPy-modified FA conjugate **s16**. After purification using the NAP-5 column, the FA-sense strand_5'ssCCMs conjugates (**s17**, **s18**, and **s19**) were obtained as previously described. FA–siRNA_5'ssCCMs (**4, 6** and **8**) were prepared from the annealing of each conjugate (**s17**, **s18** and **s19**) with the antisense strand as previously described. The purity of the desired conjugates was determined using SEC.

<u>Preparation of FA–siRNA-conjugated GALA using a branched linker at the 3’ end of the sense strand (**s25**)</u>

Scheme S5. Preparation of FA–siRNA modified at the 3' end of the antisense strand using a branched linker.

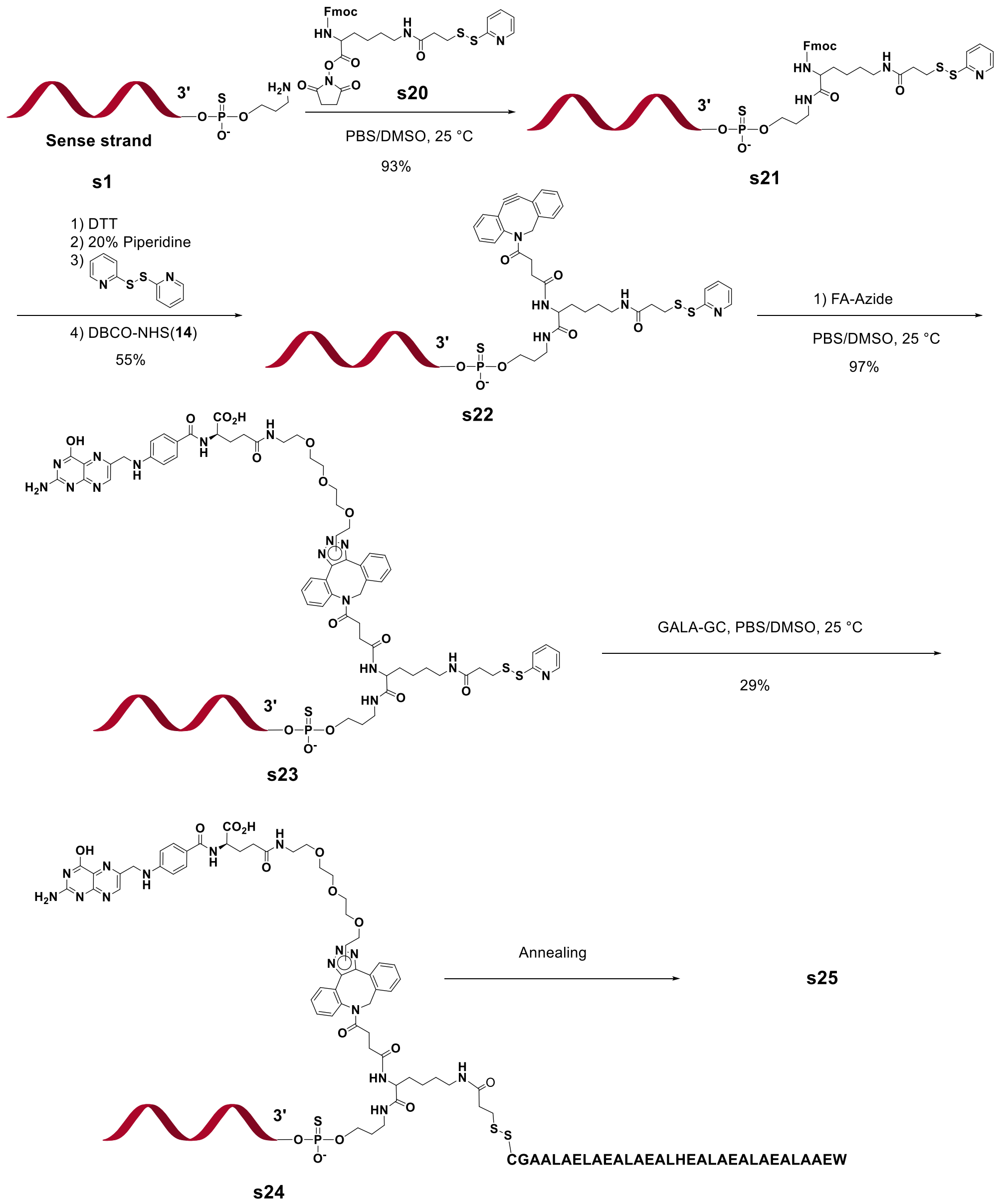

The reaction of conjugate **s1** with **s20** in PBS/DMSO at 25 °C for 3 h yielded conjugate **s21**, which was separated and concentrated using NAP-10 columns and the Amicon Ultra device. Conjugate **s21** was added to 1 M DTT solution in PBS/DMSO and left at 25 °C for 10 min before adding 20% piperidine to the DMF solution to yield the deprotected sense stand (thiol and amino groups with the branched linker-modified sense strand). This conjugate was separated and concentrated as described above. The crude branched linker-modified sense strand was added to di(2-pyridyl) disulfide in PBS/DMSO at 25 °C for 3 h to yield SPy-protected thiol. The modification of amino groups using DBCO-NHS yielded conjugate **s22**. Conjugate **s23** was obtained via an FA linker modification using a click reaction between azide and DBCO, performed as described for the preparation of FA conjugate **s16**. Conjugate **s25** was prepared using the same method for peptide conjugation and annealing of the antisense strand.

General information regarding chemical synthesis

Reagents and solvents obtained from commercial suppliers were used without purification or drying unless otherwise noted. ^{1}H NMR spectra were recorded using a Bruker 400 MHz & 500 MHz spectrometer; TMS was used as an internal standard. LC–MS/MS analysis was performed on a Waters UPLC system with an SQD-2 mass detector (Single quadrupole). HPLC and CAD analyses were performed on an ACQUITY UPLC H CLASS system (Waters) under the conditions described below.

LC–MS/MS

Condition-A (LC-MS-009): Column, ACQUITY UPLC BEH C18 (1.7 µm, 2.1 × 50 mm); Mobile phase D, 0.1% FA in H_2O; Mobile phase C, 0.1% FA in acetonitrile; T/% of C, 0/3, 0.4/3, 2.5/98, 3.5/98, and 4.03/3; Flow rate, 0.6 mL/min; Temperature, 35 °C.

Condition-B (LC-MS-007): Column, ACQUITY UPLC BEH C18 (1.7 µm, 2.1 × 50 mm); Mobile phase A, 0.1% FA in acetonitrile; Mobile phase B, 0.1% FA in water; Gradient % of B, 0/97, 0.3/97, 2.7/2, 3.5/2, and 3.51/97; Flow rate, 0.6 mL/min; Temperature, 35 °C.

Synthesis of compound **s20**

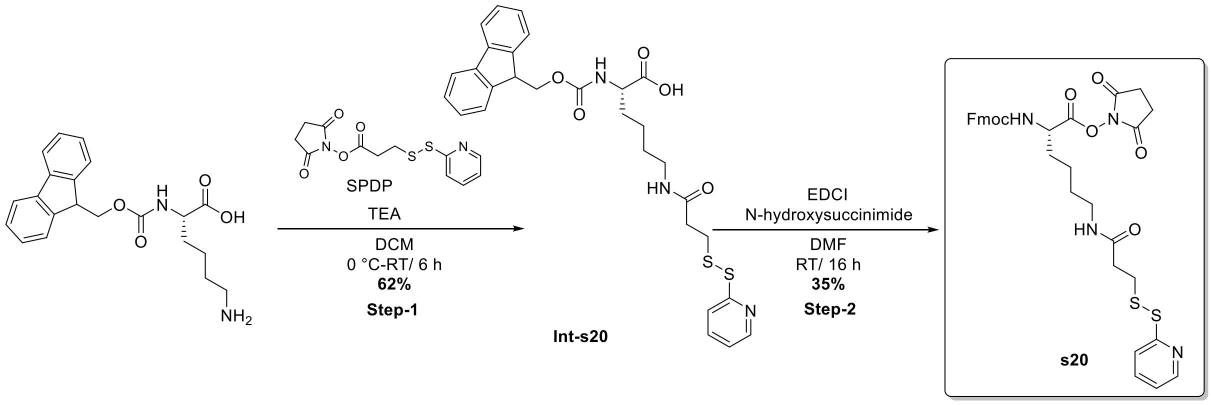

N2-(((9*H*-fluoren-9-yl)methoxy)carbonyl)-*N*6-(3-(pyridin-2-yldisulfanyl)propanoyl)-L-lysine:

After stirring a solution of (((9*H*-fluoren-9-yl)methoxy)carbonyl)-L-lysine (0.5 g, 1.36 mmol, 1.0 eq.) in dichloromethane (10 mL), we added triethylamine (0.38 mL, 2.72 mmol, 2.0 eq.) at 0 °C with continued stirring at the same temperature for 5 min before adding 2,5-dioxopyrrolidin-1-yl 3-(pyridin-2-yldisulfanyl)propanoate (0.423 g, 1.36 mmol, 1.0 eq.) at 0 °C. The reaction mixture was stirred for another 6 h at room temperature. The progress of the reaction was monitored via thin layer chromatography (TLC). After completion of the reaction, the reaction mixture was evaporated under reduced pressure to obtain a crude compound, which was purified via reversed-phase column chromatography using a W.R. Grace purification system and eluted with 60–70% acetonitrile/0.1% formic acid in water. The obtained compound was lyophilized to yield **Int-s20** (0.48 g, Y: 62%), an off-white solid.

^{1}H NMR (500 MHz, DMSO-d_6) δ ppm 1.25–1.41 (m, 4H), 1.59–1.70 (m, 2H), 2.47–2.49 (m, 2H), 2.99–3.05 (m, 4H), 3.27–3.29 (m, 1H), 3.88–3.91 (m, 1H), 4.20–4.28 (m, 3H), 7.22–7.25 (m, 1H), 7.33 (t, *J* = 7.48 Hz, 2H), 7.42 (t, *J* = 7.48 Hz, 2H), 7.58 (d, *J* = 7.93 Hz, 1H), 7.72–7.76 (m, 2H), 7.80-7.83 (m, 1H), 7.89 (d, *J* = 7.32 Hz, 2H), 7.94 (t, *J* = 5.49 Hz, 1H), 8.44–8.46 (m, 1H), 12.60 (brs, 1H). LC-MS: **Condition-A**, *m/z* 566.63 (M+H).

2,5-Dioxopyrrolidin-1-yl N2-(((9*H*-fluoren-9-yl)methoxy)carbonyl)-*N*6-(3-(pyridin-2-yldisulfanyl)propanoyl)-*L*-lysinate:

After stirring a solution of **Int-s20** (0.4 g, 0.71 mmol, 1.0 eq.) and *N*-hydroxysuccinimide (0.121 g, 1.06 mmol, 1.5 eq.) in *N, N*-dimethylformamide (5 mL), we added 3-(3-dimethylaminopropyl)-1-ethyl-carbodiimide hydrochloride (0.203 g, 1.06 mmol, 1.5 eq.) at room temperature and continued stirring the reaction mixture at the same temperature for 16 h. The progress of the reaction was monitored via TLC. After completion of the reaction, the reaction mixture was quenched with ice-cold water and extracted with ethyl acetate (twice). The combined organic layer was washed with sodium bicarbonate solution and brine, dried over anhydrous Na_2SO_4, filtered, and evaporated under reduced pressure to obtain a crude compound. The crude compound was purified via silica gel (100–200 mesh) column chromatography and eluted with 80-100% ethyl acetate/pet ether. The obtained compound was triturated with n-pentane and diethyl ether and lyophilized to yield compound **s20** (0.165 g, Y: 35%), a white solid.

^{1}H NMR (500 MHz, DMSO-d_6) δ ppm 1.42 (brs, 4H), 1.78–1.84 (m, 2H), 2.47–2.49 (m, 2H), 2.81 (brs, 4H), 2.99–3.04 (m, 4H), 4.24 (t, *J* = 7.00 Hz, 1H), 4.32–4.40 (m, 3H), 7.23 (dd, *J* = 6.87, 5.34 Hz, 1H), 7.33 (t, *J* = 7.48 Hz, 2H), 7.42 (t, *J* = 7.48 Hz, 2H), 7.70–7.72 (m, 2H), 7.75 (d, *J* = 7.93 Hz, 1H), 7.82 (td, *J* = 7.71, 1.68 Hz, 1H), 7.87–7.95 (m, 3H), 8.09 (d, *J* = 7.63 Hz, 1H), 8.45 (d, *J* = 3.97 Hz, 1H).

^{13}C NMR (100 MHz, DMSO-d_6) δ ppm 23.0, 23.5, 25.7, 26.0, 29.1, 30.9, 35.1, 47.1, 54.2, 66.1, 119.6, 120.6, 121.6, 125.8, 127.5, 128.1, 138.3, 141.2, 144.3, 150.0, 156.6, 159.7, 170.0, 173.2, 174.4.

(One peak, which was assumed to be an impurity, was observed)

LC-MS: **Conditions-B**, *m/z* 663.39 (M+H).

Analytical Data

LC-MS analysis of CCM-oligonucleotides

Oligonucleotides were analyzed using an Agilent Infinity 1260 LC–MS system with the following LC parameters: Column, ACQUITY UPLC Oligonucleotide BEH C18 (130Å, 1.7 µm, 2.1 x 50 mm, Waters); Buffer A, 8.6 mM TEA/100 mM HFIP; Buffer B, MeCN; Program, linear gradient of 10–90% buffer B over 16 min followed by 10% buffer B over 4 min; Flow rate, 0.4 mL/min; Column temperature, 60 °C

Table S1. siRNA sequences used in this study.

Gene		Sequence (5′–3′)
HPRT1	Passenger	5′ - fU^mC^fCmUfAmUfGmAfCmUfGmUfAmGfAmUfUmUfU^mA^fU-3′
	Guide	5′ -pmA^fU^mAfAmAfAmUfCmUfAmCfAmGfUmCfAmUfAmGfGmA^fA^mU-3′
B2M	Passenger	5′ - fA^mG^fGmAfCmUfGmGfUmCfUfUmUfCmUfAmUfCmU^fC^mU-3′
	Guide	5′ -fA^mG^fAmGfAmUfAmGfAmAmAfGmAfCmCfAmGfUmCfCmU^fU^mG-3′

mN and fN indicate 2′-O-methyl (2′-OMe) and 2′-deoxy-2′-fluoro (2′-F) sugar modifications, respectively, to adenosine (A), cytidine (C), guanosine (G), and uridine (U); ^ and p indicate a phosphorothioate linkage and 5′ phosphate, respectively.

Table S2. Mass spectroscopic analysis of terminal-modified oligonucleotides.

Conjugate	Mass		UV purity (%)*
	Calculated	Observed	
**s3**	7264	7263	ND
**s5**	7906	7906	94
**s5-B2M**	7897	7895	95
**s6**	7986	7987	ND
**s7**	8033	8033	ND
**s8**	11 178	11 179	86
**s9**	11 112	11 114	100
**s9-B2M**	11 101	11 102	99
**s10**	10 614	10 615	97
**s11**	10 699	10 700	98
**s12**	8496	8497	100
**s12-B2M**	8485	8485	100
**s13**	7835	7836	ND
**s13-B2M**	7824	7824	99
**s14**	7189	7186	ND
**s15**	7476	7477	ND
**s16**	8228	8227	ND
**s17**	11 307	11 309	99
**s18**	10 810	10 809	96
**s19**	10 896	10 894	96
**s21**	7524	7525	ND
**s22**	7589	7590	ND
**s23**	8231	8231	ND
**s24**	11 312	11 313	100

*Purity was not determined for intermediates (**s3**, **s5-B2M**, **s6**, **s7**, **s13**, **s14–s16**, and **s21–s23**).

Table S3. Purity of FA–siRNA derivatives.

Conjugate	No		%UV Purity
**1**	Passenger	**s5-ssHPRT1**	97
	Guide	Unconjugated antisense strand	
**1-siB2M**	Passenger	**s5-ssB2M**	97
	Guide	Unconjugated antisense strand	
**2**	Passenger	**s5-ssHPRT1**	98
	Guide	**s8-asHPRT1**	
**3**	Passenger	**s5-ssHPRT1**	95
	Guide	**s9-asHPRT1**	
**3-siB2M**	Passenger	**s5-ssB2M**	97
	Guide	**s9-asB2M**	
**4**	Passenger	**s17-ssHPRT1**	97
	Guide	Unconjugated antisense strand	
**5**	Passenger	**s5-ssHPRT1**	94
	Guide	**s10-asHPRT1**	
**6**	Passenger	**s18-ssHPRT1**	90
	Guide	Unconjugated antisense strand	
**7**	Passenger	**s5-ssHPRT1**	92
	Guide	**s11-asHPRT1**	
**8**	Passenger	**s19-ssHPRT1**	91
	Guide	Unconjugated antisense strand	
**9**	Passenger	**s5-ssHPRT1**	98
	Guide	**s12-asHPRT1**	
**9-siB2M**	Passenger	**s5-ssB2M**	99
	Guide	**s12-asB2M**	
**10**	Passenger	**s5-ssHPRT1**	93
	Guide	**s13-asHPRT1**	
**10-siB2M**	Passenger	**s5-ssB2M**	99
	Guide	**s13-asB2M**	
**s25**	Passenger	**s24-ssHPRT1**	94
	Guide	Unconjugated antisense strand	

*Purity of conjugates determined via SEC-HPLC.

A

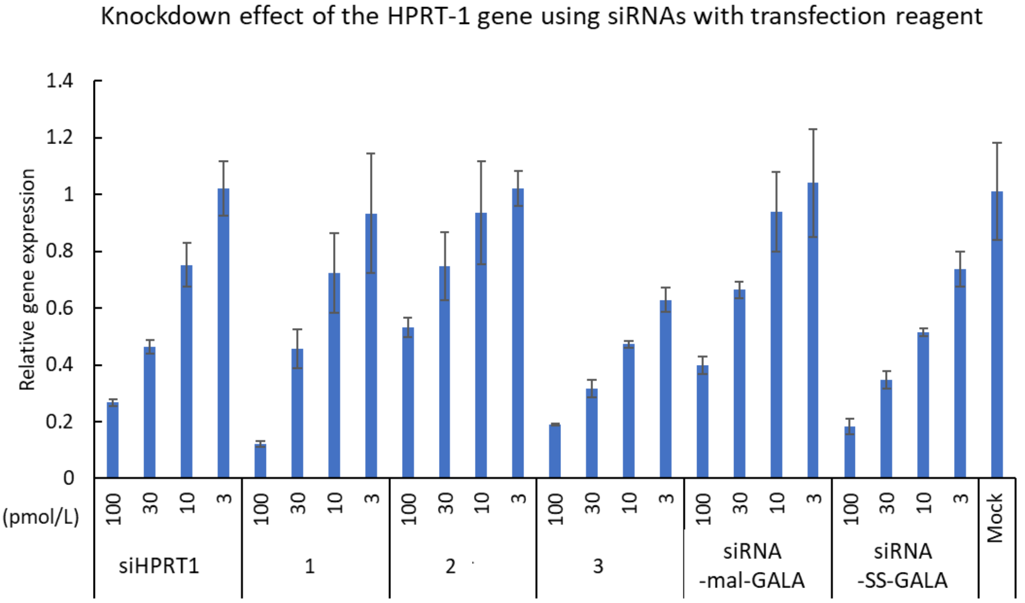

B

compound	IC_{50} value (pmol/L)
siHPRT1	26.1
**1**	25
**2**	>100
**3**	8.1
**siRNA-mal-GALA**	63.1
**siRNA-SS-GALA**	11

C

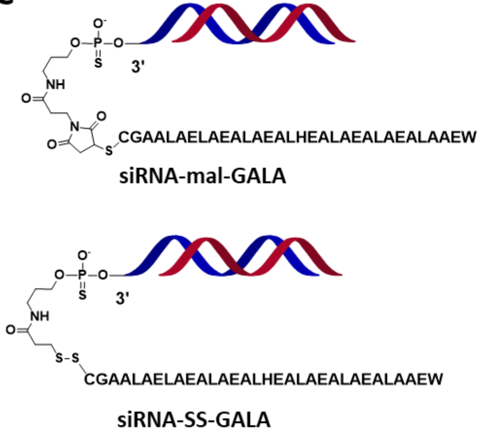

Figure S1. The gene knockdown efficiency of siRNA and conjugates. A) The gene silencing activities in KB cells. The conjugates and siRNA with the transfection reagent were added to cells for 3 days. HPRT-1 mRNA expression was measured via quantitative PCR and normalized to that of ACTB mRNA. B) The IC50 value of the siRNA and conjugates. C) The structure of GALA-conjugated siRNAs. The results of *in vitro* experiments are expressed as the mean ± SD of triplicate experiments.

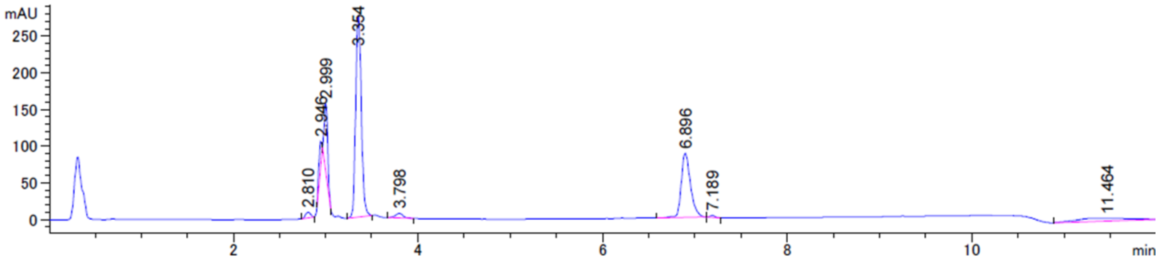

RT (min)_LC	Molecular Weight	Estimation
2.946	7922.83	**s9 without GALA**
	8228.06	**s9** ; GALA exchanged by GSH
3.354	7905.04	**s5**
6.896	11113.42	**s9**

Figure S2. Stability of compound 3 with a disulfide linker in the reduction condition. The stability of the disulfide linker of conjugate **3** was tested in the reduction condition. Conjugate **3** was mixed with GSH (Reduced form), and the reduction reaction was monitored via liquid chromatography–tandem mass spectrometry (LC–MS/MS) at 22 hr. Each peak of conjugate **3**, including **s9**, **s5**, and its expected degradation products, were assigned by their masses.

A

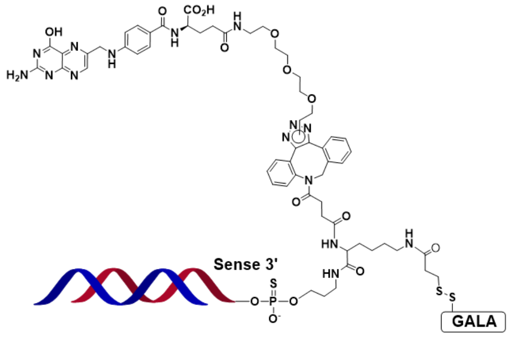

FA-siRNA_ss3'_br_GALA

B

Conjugate	K_D (nmol/L)
FA-siRNA_ ss3'_br_GALA	0.162

C **D**

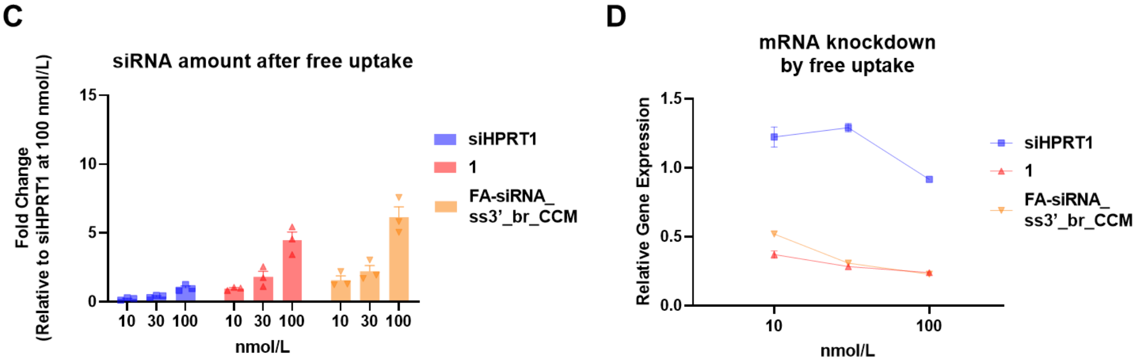

Figure S3. Properties of FA–siRNA modified at the 3' end of the antisense strand using a branched linker. A) Structure of modified FA–siRNA (**s25**). B) Binding properties of FA–siRNA and its derivatives to human folate receptor 1 (hFOLR1). C) Gene KD efficiency of FA–siRNA_ss3'_br_GALA. *HPRT1* mRNA expression was quantified via RT-qPCR and normalized to *ACTB* mRNA expression at 3 d after treatment. D) The amount of siRNA after free uptake in KB cells. Each oligonucleotide was added to KB cells for 1 d. Cellular uptake was analyzed via stem-loop RT-qPCR. Data are expressed as the mean ± SE from triplicate experiments.

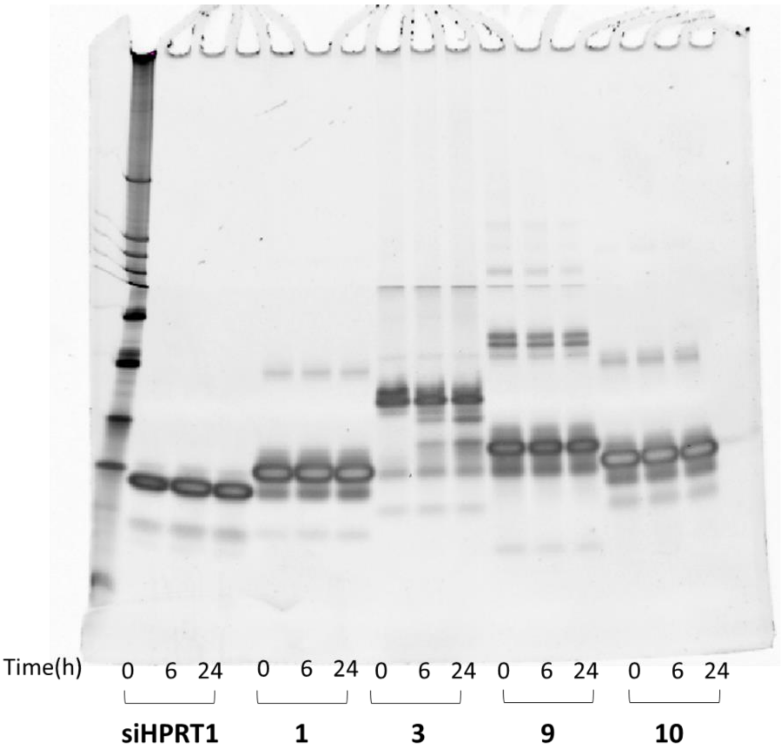

Figure S4. The evaluation of stability of the conjugates using snake venom phosphodiesterase (SVPD).

The stability of FA-siRNA and conjugates were evaluated by PAGE using SVPD. The siHPRT1 and conjugates were incubated in a final concentration of 2 μmol/L. The SVPD in 50 mM Tris (pH 7.5), 8 mM MgCl2 was added to each conjugate solution in a final concentration of 3.75 mU/mL. After the incubation for 0, 6, and 24 h, reaction was stopped by adding EDTA and heating at 95 ℃ for 10 min, then PAGE analysis was conducted.

A

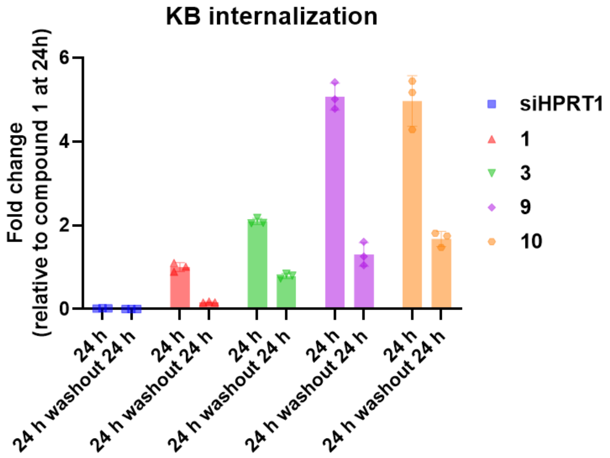

B

Treatment	Fold change relative to 1 at 24h		Accumulation ratio of each
	24h	48h	24h/48h
**siHPRT1**	0.017	0.0028	0.17
**1**	1.0	0.17	0.17
**3**	2.1	0.79	0.38
**9**	5.1	1.3	0.26
**10**	5.0	1.7	0.34

Figure S5. The stability of the conjugates and fully chemically modified siRNA.

A) The stability of FA-siRNA and conjugates. After 24 h of incubation, the cells were washed and incubated for another 24 h. The remaining siRNA and conjugate in KB cells were quantified via stem-loop RT-qPCR. The results are expressed as the mean ± SD of values from triplicate experiments. B) The comparison of remaining siRNA at 24 and 48 h. The amount of cellular siRNA at 24 h was used as the initial value. The stability of the conjugates was evaluated via one-way analysis of variance, followed by Dunnett's test, compared to that of compound 1 (*p < 0.001).

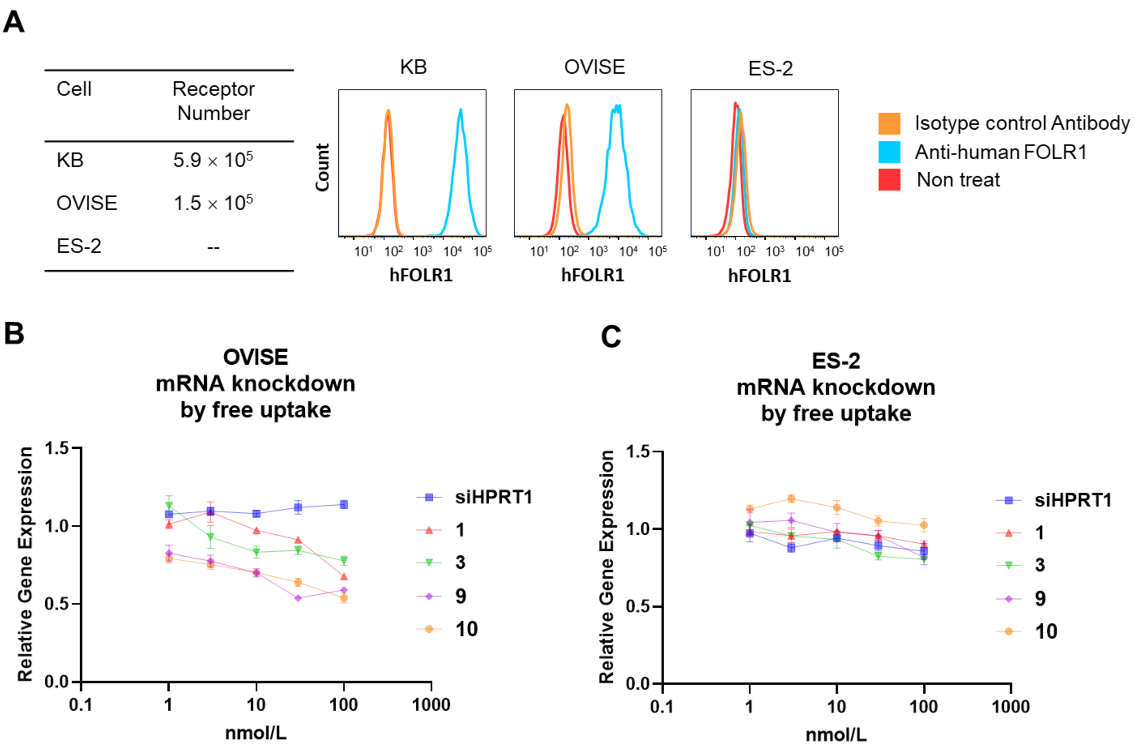

Figure S6. KD efficiency of FA–siRNA modified at the 3′ end of the antisense strand in various cells. A) hFOLR1 expression in KB, OVISE, and ES-2 cells analyzed via flow cytometry. B) KD activity in OVISE cells using FA-siRNA conjugates. C) KD activity in ES-2 cells using FA-siRNA conjugates. *HPRT1* mRNA expression was quantified via RT-qPCR and normalized to *ACTB* mRNA expression at 3 d after treatment.

Conjugate s5-ssHPRT1

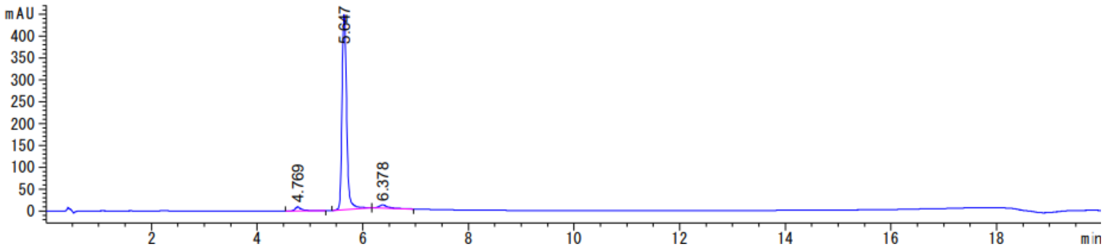

Conjugate s5-ssB2M

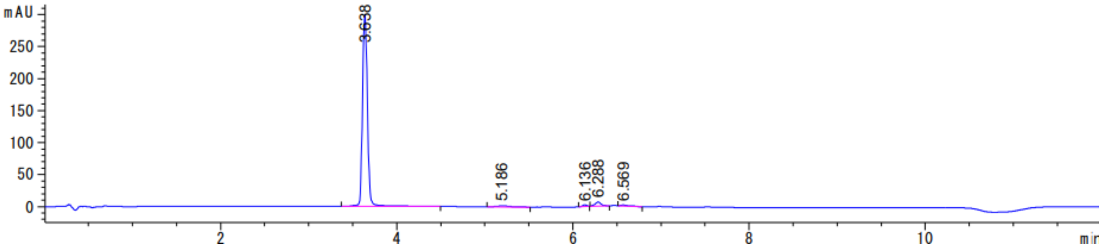

Conjugate s8-asHPRT1

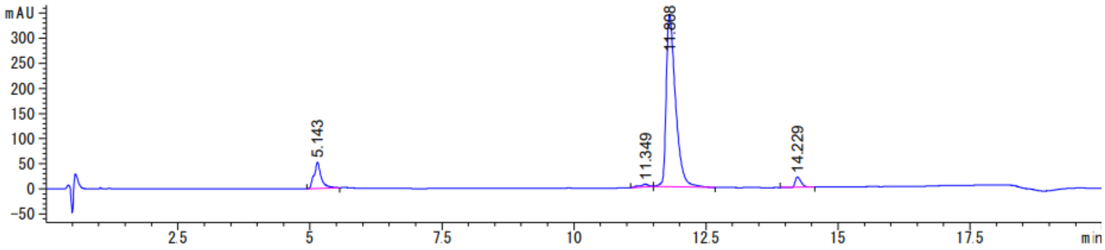

Conjugate s9-asHPRT1

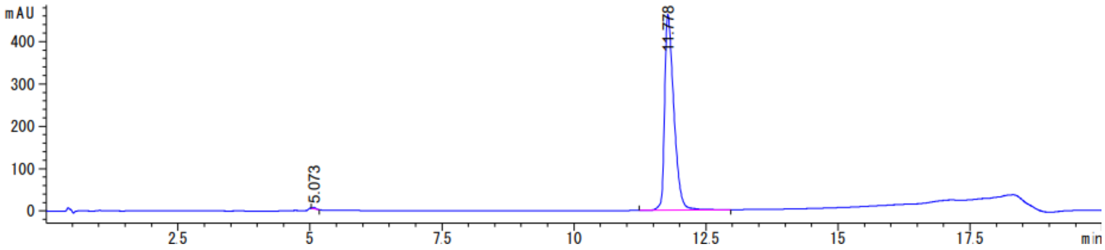

Conjugate s9-asB2M

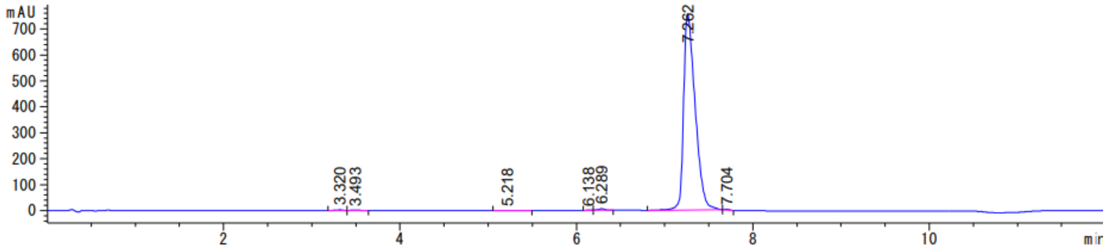

Conjugate s10-asHPRT1

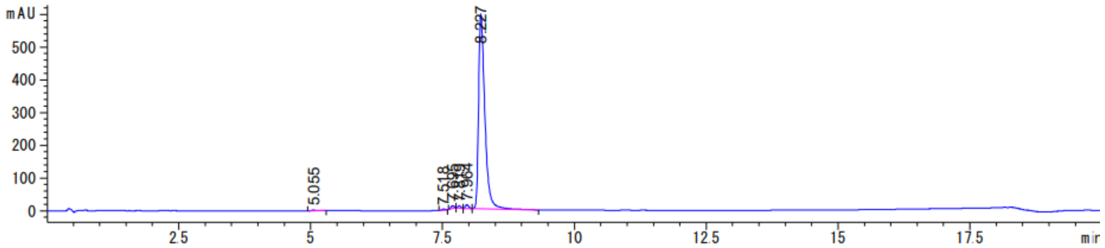

Conjugate s11-asHPRT1

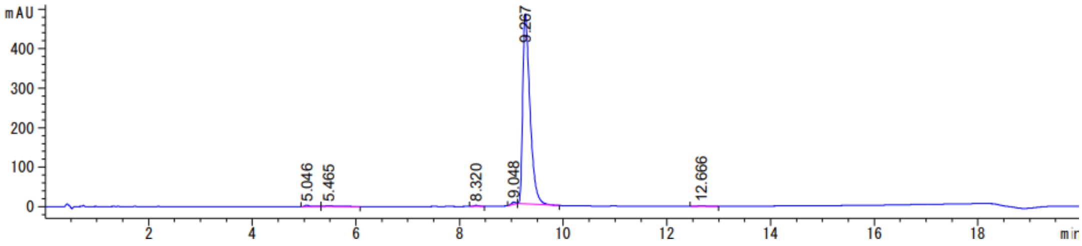

Conjugate s12-asHPRT1

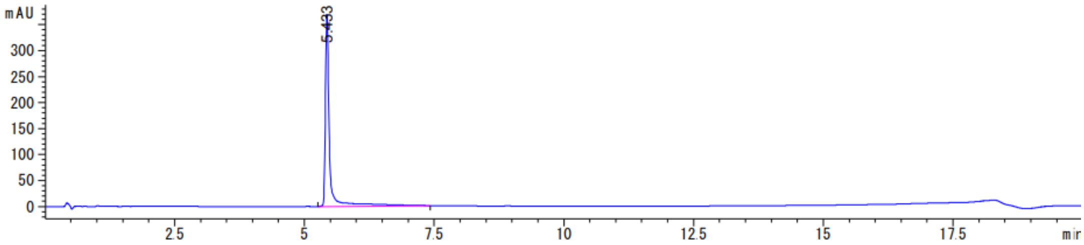

Conjugate s12-asB2M

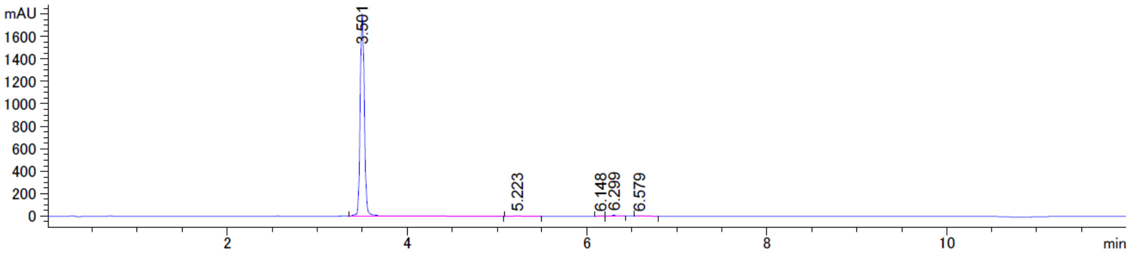

Conjugate s13-asB2M

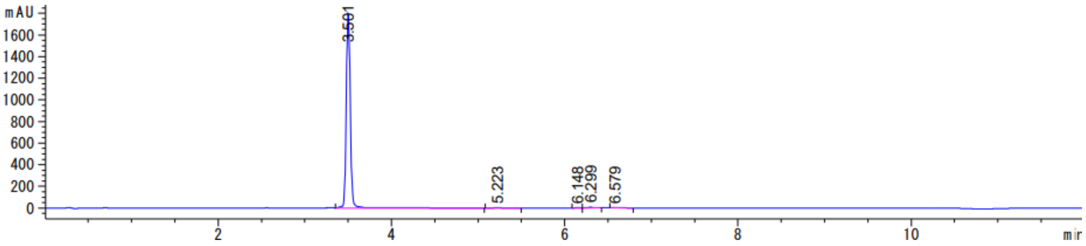

Conjugate s17-ssHPRT1

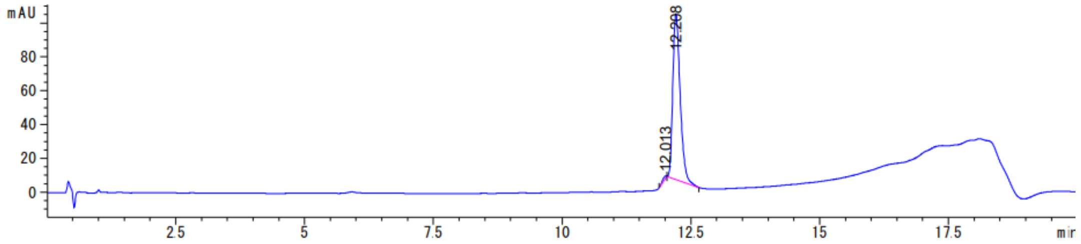

Conjugate s18-ssHPRT1

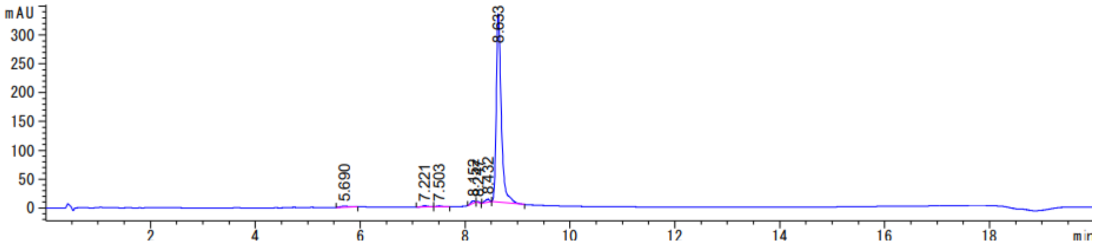

Conjugate s19-ssHPRT1

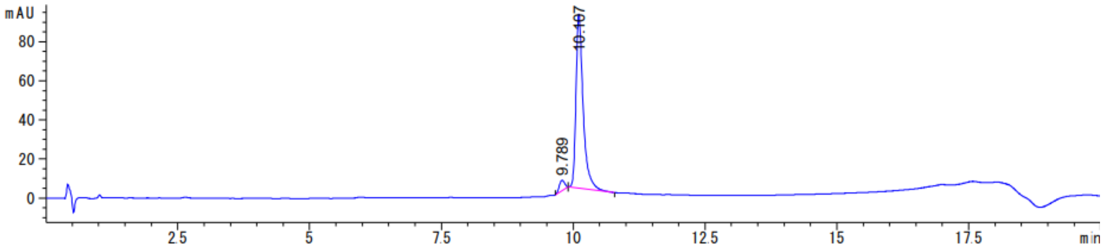

Conjugate s24-ssHPRT1

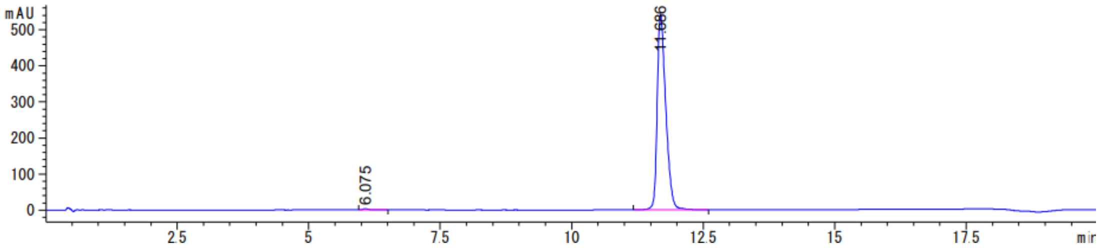

SEC analysis of siRNA conjugates

Conjugate 1

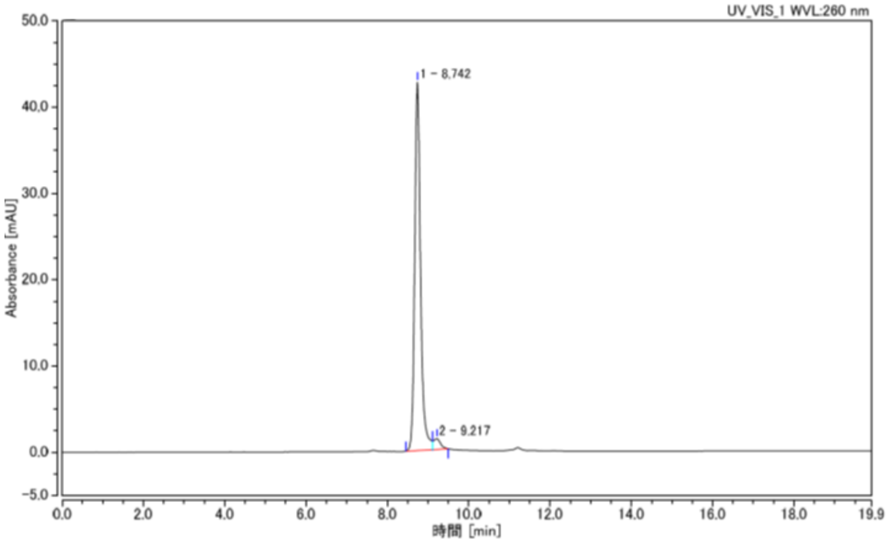

Conjugate 1-siB2M

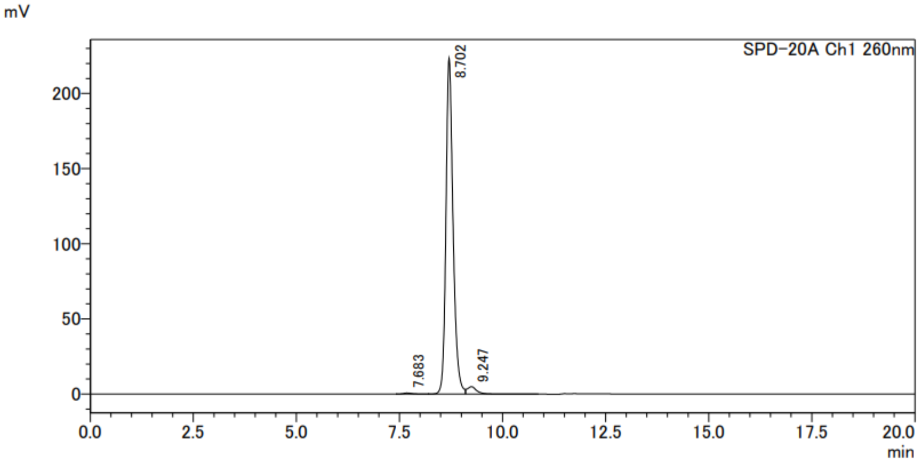

Conjugate 2

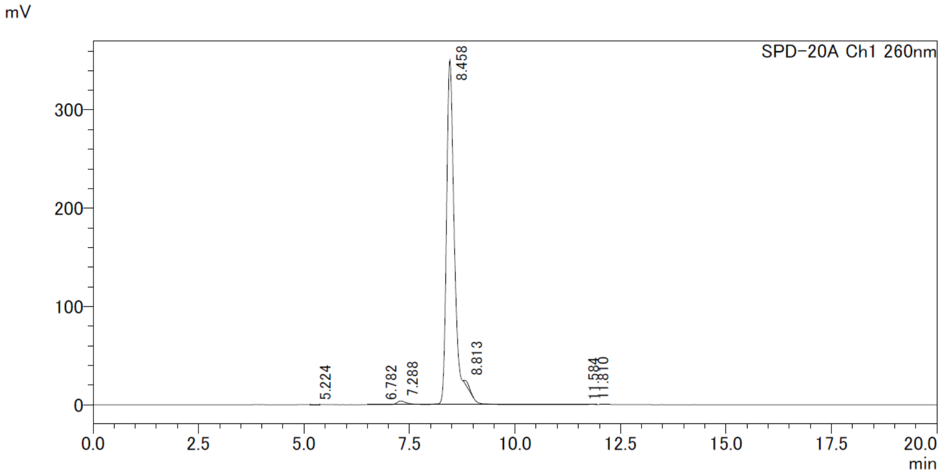

Conjugate 3

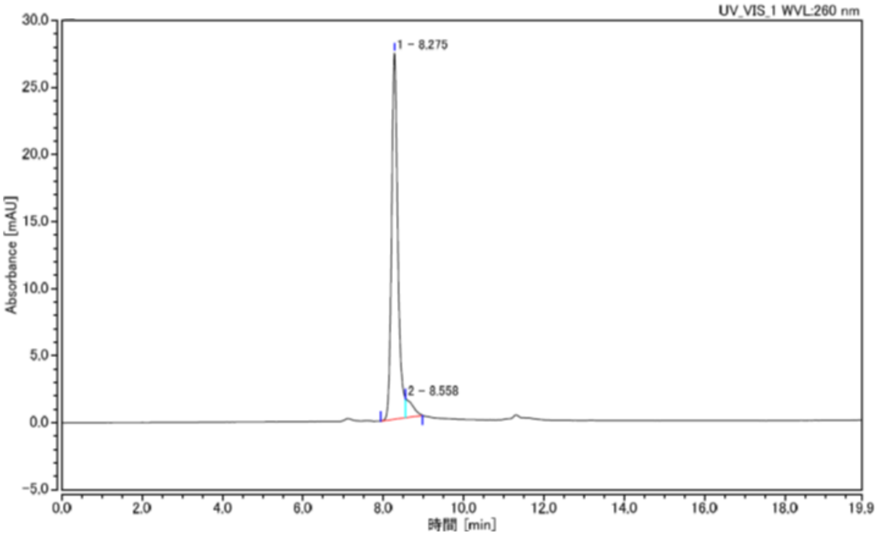

Conjugate 3-siB2M

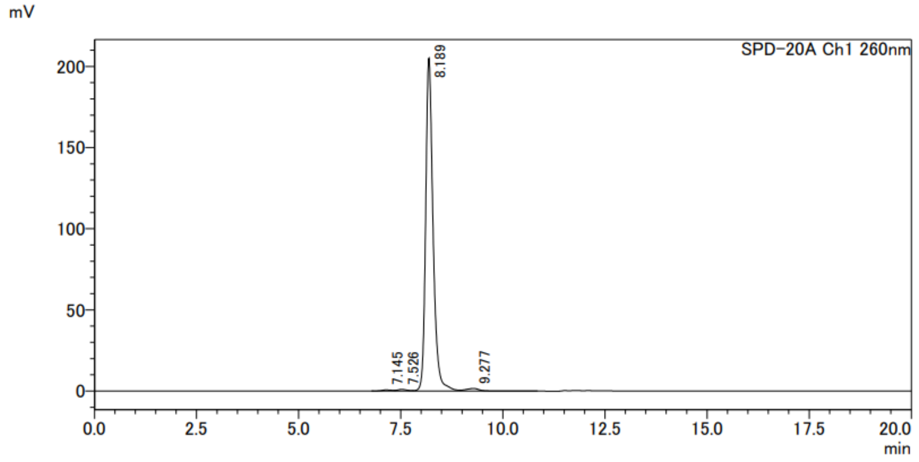

Conjugate 4

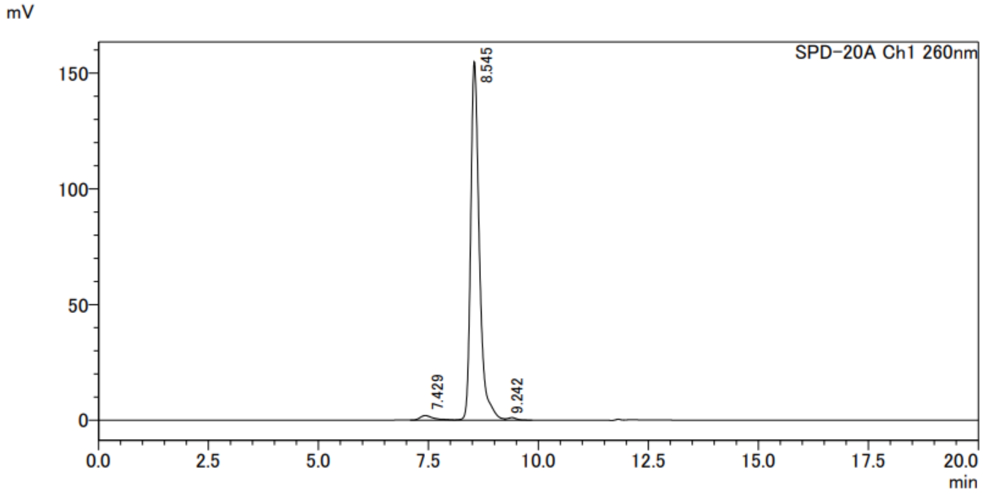

Conjugate 5

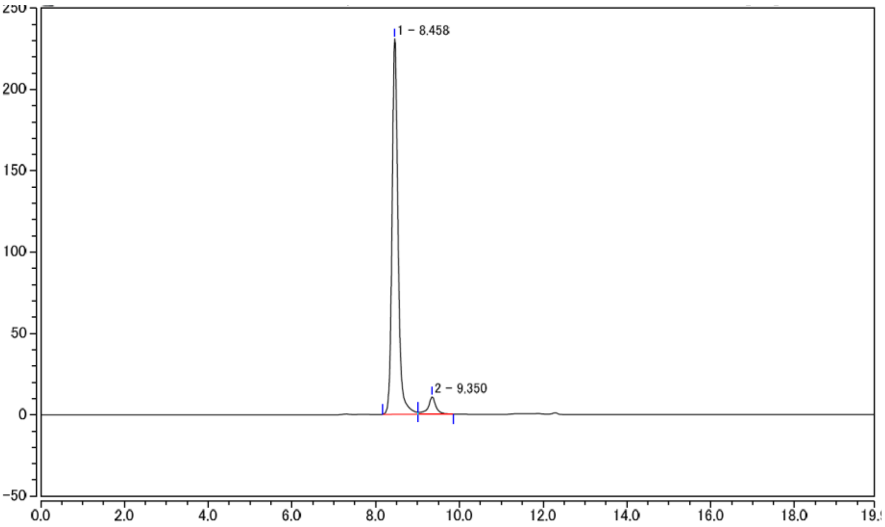

Conjugate 6

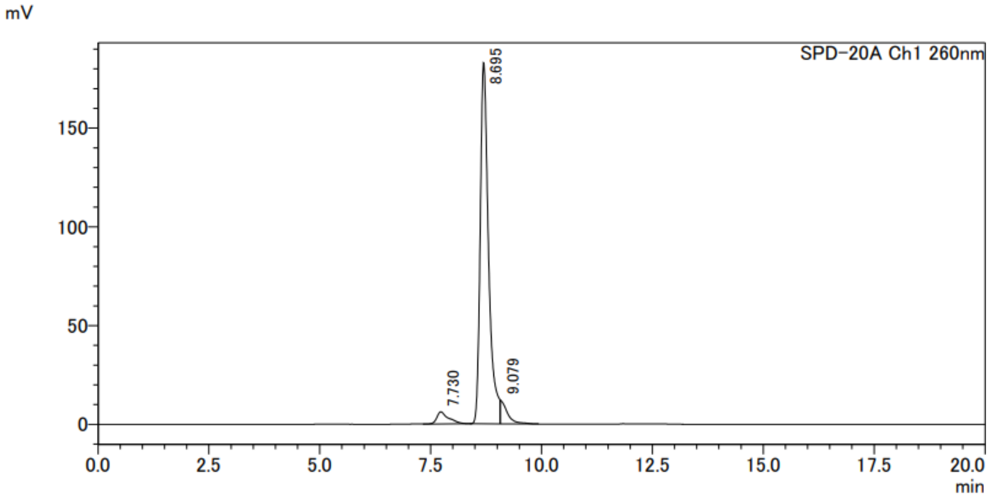

Conjugate 7

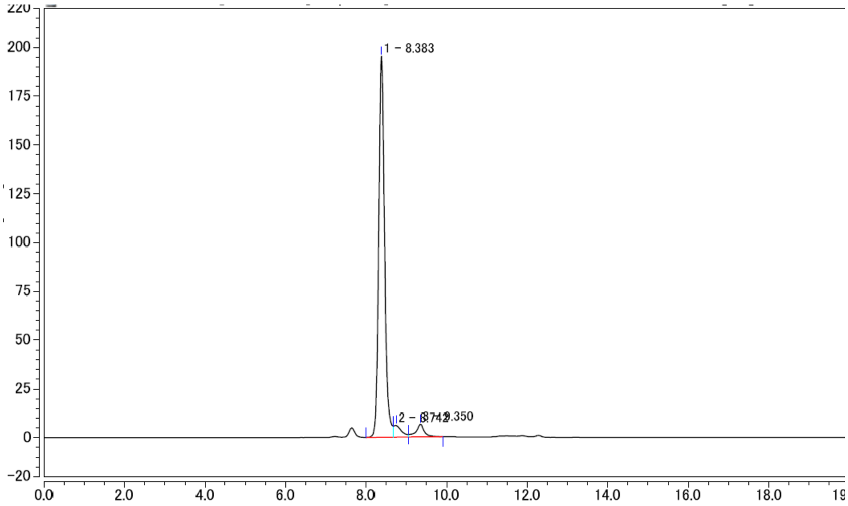

Conjugate 8

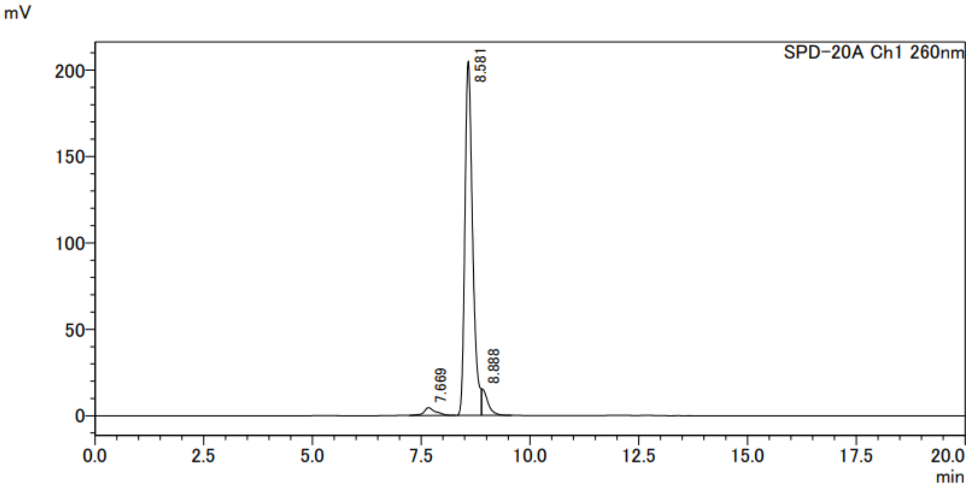

Conjugate 9

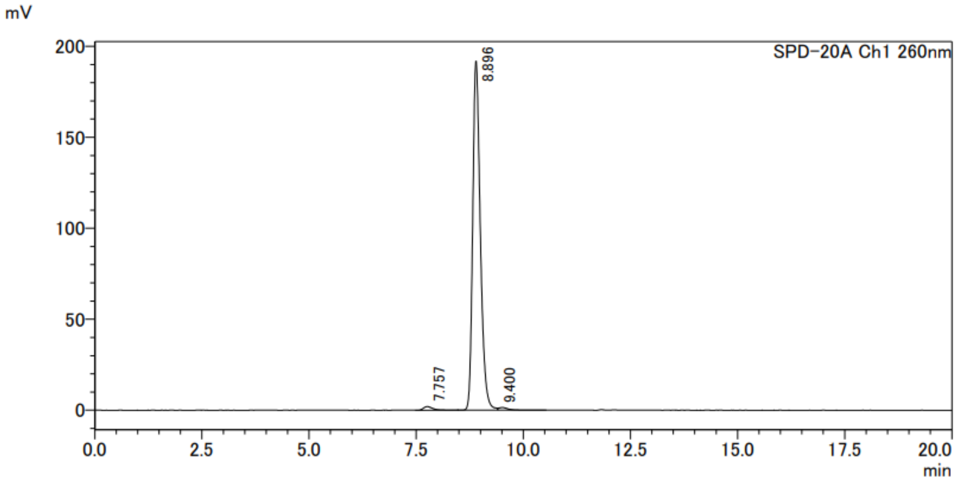

Conjugate 9-siB2M

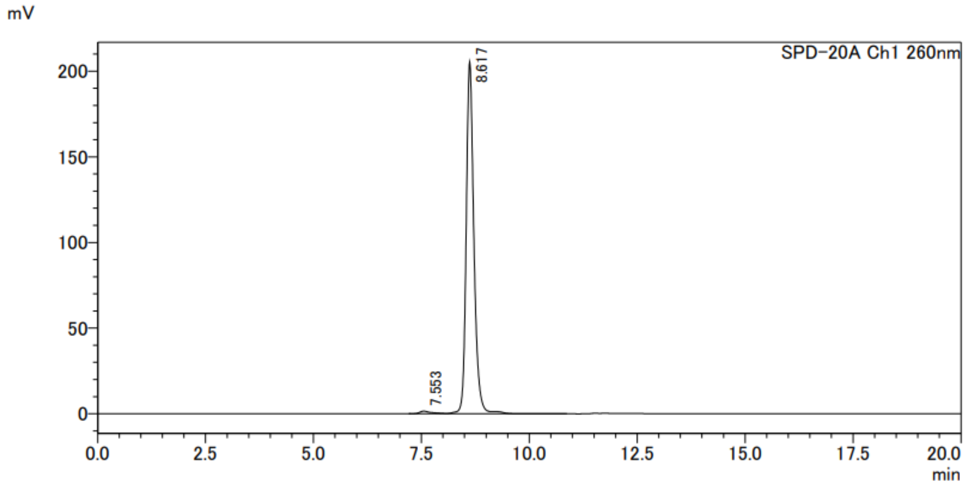

Conjugate 10

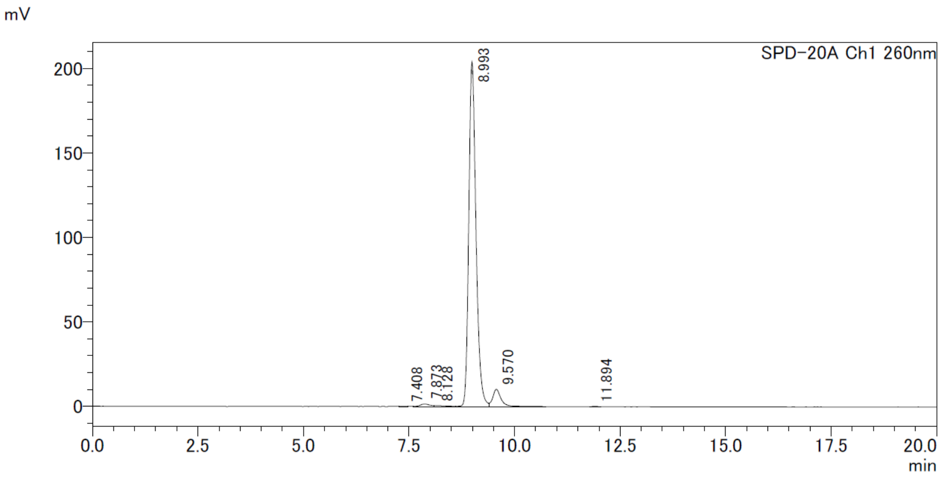

Conjugate 10-siB2M

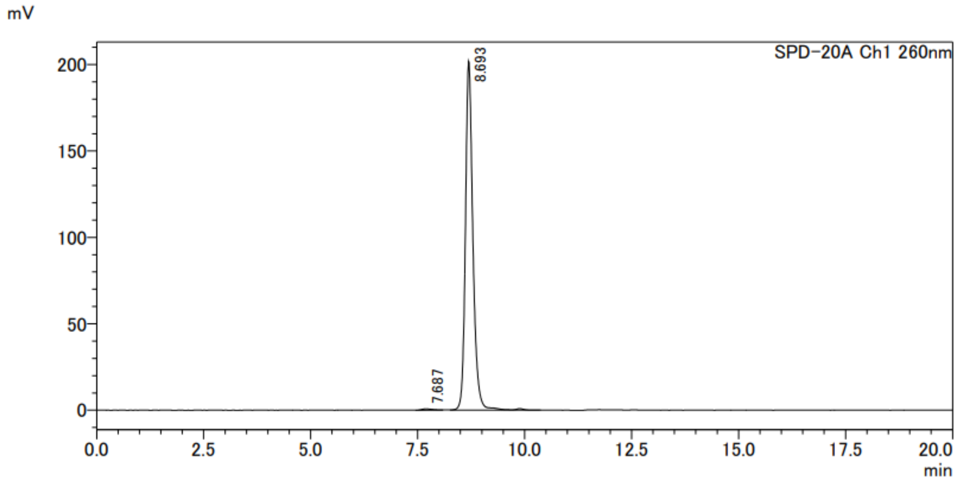

Conjugate s25

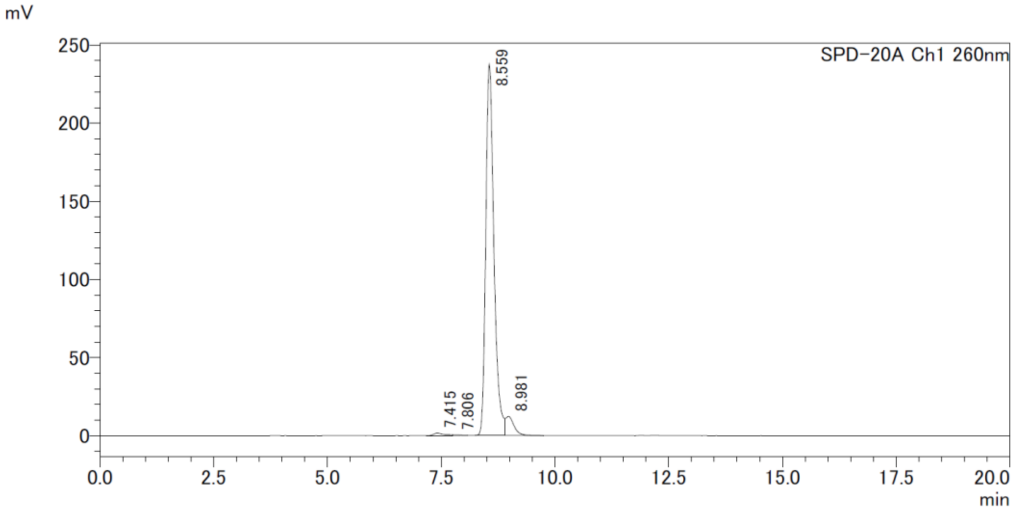

Supplemental References

1. Singh, Y.; Murata, P.; Defrancq, E. Recent developments in oligonucleotide conjugation. *Chem. Soc. Rev.* **2010**, 39, 2054–2070.
